# Single-guide RNA Cas9 and enhanced-deletion Cas9 rescue a recurrent *USH2A*-related splicing defect

**DOI:** 10.1016/j.omtn.2025.102523

**Published:** 2025-03-21

**Authors:** Pietro De Angeli, Salome Spaag, Stefanida Shliaga, Arturo Flores-Tufiño, Malte Ritter, Masoud Nasri, Katarina Stingl, Laura Kühlewein, Bernd Wissinger, Susanne Kohl

**Affiliations:** 1 University Hospital Tübingen, Centre for Ophthalmology, Institute for Ophthalmic Research, 72076 Tübingen, Germany; 2 University Hospital Tübingen, Department of Oncology, Hematology, Clinical Immunology, and Rheumatology, 72076 Tübingen, Germany; 3 University Eye Hospital, Center for Ophthalmology, University of Tübingen, 72076 Tübingen, Germany

**Keywords:** MT: RNA/DNA Editing, Cas9, CRISPR, gene editing, splicing, USH2A, TREX2, VLP, indels, Usher syndrome

## Abstract

Missplicing of transcripts is a frequent molecular mechanism in a wide range of inherited genetic conditions. Therapeutic splicing correction can be achieved through antisense oligonucleotides; however, they do not enable permanent correction. Concurrently, CRISPR-Cas9 approaches often rely on dual-guide RNA-induced larger deletions—for instance, pseudoexons removal—which raises concerns about higher genotoxicity from multiple double-strand breaks. We therefore investigated single-guide RNA CRISPR-Cas9 approaches to address the recurrent pathogenic *USH2A*:c.7595-2144A>G deep-intronic variant. Using single-guide RNAs with either Cas9 or Cas9 fused to TREX2 (EDCas9), we restored correct splicing in a minigene assay and patient-derived fibroblasts. Cas9 with single-guide RNAs generated small indels, but their frequency and extent varied between models, resulting in variable productivity with respect to splicing rescue efficacy. In contrast, EDCas9 produced larger, directional deletions with a consistent profile across both models, effectively disrupting missplicing-inducing sequences and ensuring robust splicing correction. Off-target assessments revealed a safe profile for both Cas9 and EDCas9, with EDCas9 additionally preventing targeted translocations. Virus-like particles delivered EDCas9 and a lead gRNA, demonstrating suitability as a transient delivery system. In conclusion, EDCas9 emerges as a flexible and powerful editing approach for addressing the pathogenic *USH2A*:c.7595-2144A>G variant, paving the way for further therapeutic investigation.

## Graphical abstract

**Figure:**
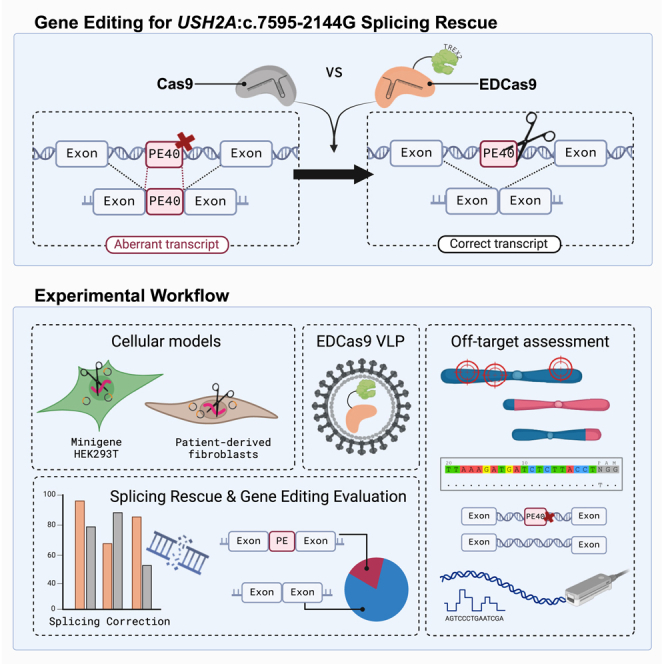

## Teaser

This study investigates single-guide RNA CRISPR-Cas9 approaches for correcting the pathogenic *USH2A*:c.7595-2144A>G splicing variant. Using Cas9 and EDCas9, the authors achieve consistent splicing correction in both minigene assays and patient fibroblasts. EDCas9 demonstrated robust efficiency, safe off-target profile, and potential for therapeutic delivery via virus-like particles.

## Introduction

Correct splicing is essential to guarantee the production of correct mRNA transcripts in eukaryotic cells, fundamental for translation into functional proteins.^1^ The process is regulated by a complex protein machinery known as a spliceosome.^2^ It recognizes *cis*-acting sequences in the pre-mRNA molecule (i.e., splicing signals), which together orchestrate the recognition of intron-exon boundaries, resulting in the removal of introns from the pre-mRNA molecule. In the last decade, an increasing number of disease-causing variants, resulting in aberrant splicing has underscored the role of such variants and aberrantly spliced transcripts as a frequent pathomechanism in inherited disorders.^3^ Additionally, the unique pathogenic molecular mechanism associated with variants impacting splicing has unveiled new avenues for the exploration of innovative therapeutic strategies. In particular, deep-intronic variants that induce pseudoexon (PE) activation can account for a considerable fraction of disease-associated variants (or alleles) in some genes.^4^^,^^5^^,^^6^

Antisense oligonucleotides (ASOs) and CRISPR-Cas9 genome editing have been harnessed to effectively interfere with aberrant splicing induced by such variants, eventually restoring regular splicing.^7^^,^^8^^,^^9^ ASOs function through masking *cis*-acting sequences on the pre-mRNA that trigger aberrant splicing, thereby preventing their recognition by the spliceosome. As a result, the formation of correct mature mRNA transcript is restored.^10^ However, in the context of therapeutic translation, splicing correction achieved by ASOs requires recurrent re-administration of the therapeutic compound and thus is incompatible with a single curative treatment.^11^

The use of CRISPR-Cas9 genome editing potentially enables permanent repair of the splicing defect. Current genome-editing approaches to rescue aberrant splicing, involving PE inclusion due to deep-intronic variants, primarily rely on the use of pairs of guide RNAs (gRNAs) targeted to generate a genomic deletion encompassing the deep-intronic variant and part or the whole PE sequence.^12^^,^^13^^,^^14^^,^^15^ However, the generation of multiple double-stranded breaks (DSBs) for therapeutic purposes arguably raises safety concerns due to higher genotoxicity.^16^^,^^17^^,^^18^ For this reason, gene-editing approaches based on single DNA breaks might be preferable. Although approaches based on single-guide RNAs (sgRNAs) designed to target sequences in close proximity to those involved in aberrant splicing have also been demonstrated, their investigation is limited to a few examples.^14^

As an alternative to paired gRNA/CRISPR-Cas9 approaches, we have applied and validated two sgRNA/CRISPR-Cas9 approaches to rescue the splicing defect induced by the recurrent *USH2A*:c.7595-2144A>G deep-intronic variant in minigene assays and patient-derived fibroblasts. Pathogenic variants in the *USH2A* gene result in Usher syndrome, a group of autosomal recessive rare genetic disorders that affects both hearing and vision.^1^ Specifically, disease-causing *USH2A* variants account for up to ∼50% of all Usher syndrome cases, and are also a common cause of isolated non-syndromic retinitis pigmentosa.^2^^,^^3^^,^^19^^,^^20^ Although many pathogenic variants in *USH2A* are private and therefore ultra-rare, important founder variants have been identified. For example, the *USH2A*:c.7595-2144A>G deep-intronic variant is common and accounts for up to ∼4% of total *USH2A* alleles, depending on population.^4^^,^^5^^,^^6^ Its pathogenic mechanism involves the activation of a cryptic donor splice site, which leads to the retention of a 152-bp intronic sequence in the mature mRNA transcript between exons 40 and 41 (acknowledged as PE40). The presence of the PE sequence in the mRNA results in a premature termination codon (p.Lys2532Thrfs∗56), predicted to lead to the degradation of the mutant transcript via the nonsense-mediated decay (NMD) pathway.^5^^,^^7^

The first gene-editing approach, namely gRNA/Cas9, utilizes wild-type (WT) Cas9 to induce small insertions or deletions (indels) at sequences involved in the aberrant splicing process (i.e., the cryptic acceptor, donor splice sites, and/or the deep-intronic variant). The second editing strategy, namely gRNA/enhanced-deletion Cas9 (EDCas9), implements a chimeric Cas9 molecule, where Cas9 is fused to the human three prime repair exonuclease 2 (TREX2).^21^^,^^22^ The resulting molecule, EDCas9, is coupled to the same individual sgRNAs. As a result, owing to the combined activity of Cas9 and TREX2, the occurrence of deletions is notably increased, leading to a more extended perturbation of the targeted DNA sequences involved in aberrant splicing. The molecular mechanism of action is that following a single DSB generated by Cas9, TREX2 processes the resulting DNA ends, thereby promoting the generation of deletions. In both editing strategies, part of the achieved sequence perturbations prevents spliceosome recognition of the splicing defect, thereby leading to the restoration of correct splicing.^21^ Furthermore, the fusion of Cas9 to TREX2 has been reported to decrease chromosomal translocation, large-scale chromosomal deletions, and, when delivered by adeno-associated virus (AAV) particles, low AAV genome integration events at the targeted site. These features significantly enhance the safety profile of this gene-editing approach.^23^^,^^24^

## Results

### Design of sgRNA gene-editing strategies to rescue the *USH2A*:c.7595-2144A>G-induced splicing defect

**Figure 1:**
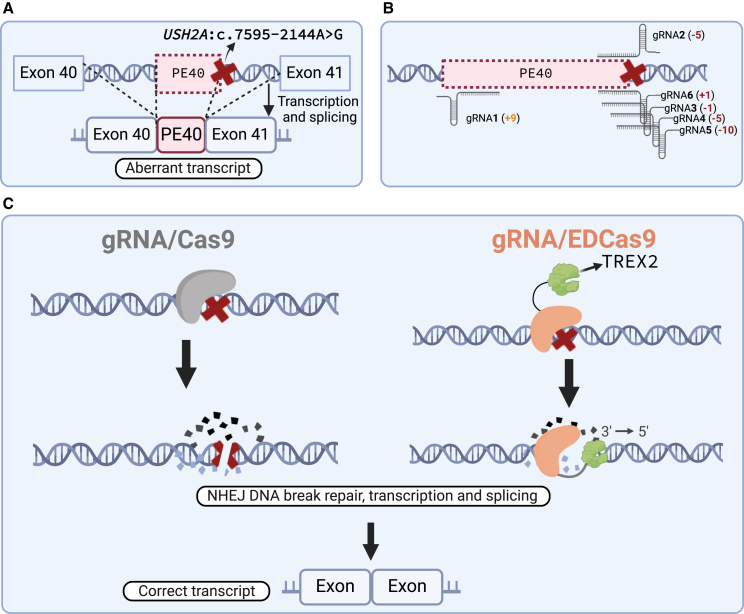
Schematic overview of gRNA/Cas9 and gRNA/EDCas9 for *USH2A*:c.7595-2144A>G splicing defect rescue (A) The deep-intronic variant *USH2A*:c.7595-2144A>G (marked by a red X) mediates a splicing defect in which a 152-bp intronic sequence, also known as pseudoexon 40 (PE40), is included in the final mRNA transcript of *USH2A* during transcription and splicing. This PE insertion results in a frameshift in the open reading frame, leading to the formation of a premature termination codon. As a result, the transcript is expected to be degraded by the nonsense-mediated mRNA decay pathway. (B) Relative locations of the sgRNAs used within this research in relation to the *USH2A*:c.7595-2144A>G deep-intronic variant. The exact nucleotide position of the gRNA cleavage site in relation to *USH2A*:c.7595-2144A>G is given in parentheses. (C) Illustration of the sgRNA/Cas9 and/EDCas9 mechanisms leading to splicing rescue. The gRNA/Cas9 complex mediates double-stranded break formation, which is repaired by non-homologous end joining (NHEJ) leading to targeted insertions or deletions. For gRNA/EDCas9, following double-stranded break, the two ends of the target strand and the 5′ end of the non-target strand are firmly engaged with Cas9. Hence, the fusion partner TREX2 further processes the non-target strand in the 3′ to 5′ direction, resulting in “enhanced deletions” biased toward this direction. In both cases, the resulting perturbation of the sequences involved in aberrant splicing, prevents the spliceosome from recognizing the faulty splicing signals, thereby reverting to correct splicing.

To target the splicing defect resulting in the retention of the 152-bp PE (Figure 1A), six different sgRNAs were designed (Figure 1B): gRNA1 is located upstream of the cryptic acceptor site and five (gRNA2, gRNA3, gRNA4, gRNA5, and gRNA6) target the cryptic donor splice site induced by *USH2A*:c.7595-2144A>G. Four out of six sgRNA sequences overlap with the deep-intronic variant (gRNA3, gRNA4, gRNA5, and gRNA6), while in the case of gRNA2, the deep-intronic variant is within the protospacer adjacent motif sequence. Upon DSB, Cas9 strongly binds three out of four strand ends at the generated break (Figure 1C). These include the 5′ and 3′ ends on the target strand and the 5′ end on the non-target strand, while the 3′ end of the non-target strand is quickly released.^25^ Owing to this mechanism, we speculated that the release prioritizes the processing of the 3′ end of the non-target strand, leading to the generation of deletions biased toward this direction. To empower the potential directional bias of EDCas9, all gRNAs (besides gRNA2) are designed to prompt deletions toward sequences involved in the aberrant splicing.

### Cas9- and EDCas9-mediated splicing rescue using a *USH2A*:c.7595-2144A>G minigene model

**Figure 2:**
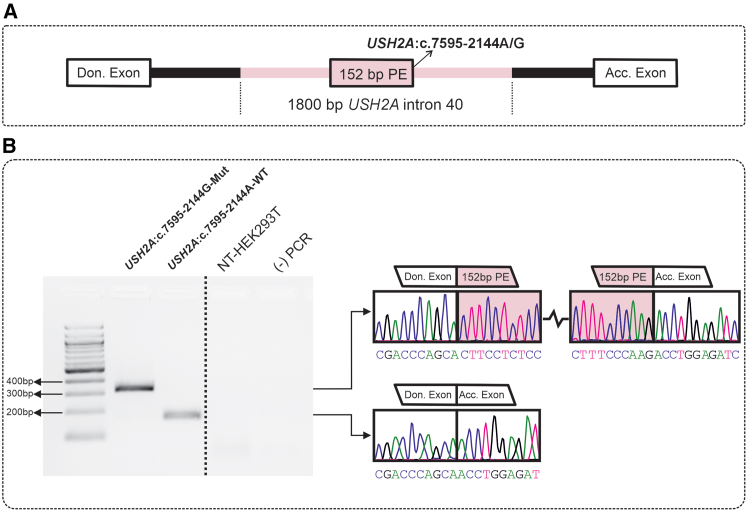
*USH2A*:c.7595-2144A>G-induced splicing defects induced by minigene assay in HEK293T (A) Graphical representation of the *USH2A* minigene plasmid cloned in the splicing-exon trap pSPL3 backbone plasmid, highlighting the position of the 152-bp PE retained upon missplicing. Don. Exon, donor exon; Acc. Exon, acceptor exon. (B) Minigene splicing pattern for the *USH2A*:c.7595-2144A>G deep-intronic variant in HEK293T. The agarose gel displays PCR amplicon bands from splicing outcomes of mutant *USH2A* (*USH2A*:c.7595-2144G-Mut) and wild-type minigene (*USH2A*:c.7595-2144G-WT) plasmids. Non-transfected HEK293T (NT-HEK293T) and the no-DNA (−) PCR are shown as controls. PCR amplicon sequencing confirms the presence of the 152-bp PE sequence in the mutant *USH2A* and the correctly spliced cDNA sequence in the WT minigene.

To investigate the potential splicing rescue of the designed sgRNAs coupled to EDCas9 (sgRNA/EDCas9) in comparison to Cas9 (sgRNA/Cas9), we designed (Figure 2A) and cloned a minigene plasmid that upon transfection in HEK293T recapitulates the *USH2A*:c.7595-2144A>G-induced splicing defect, hereby confirming and recapitulating the expected aberrant splicing (Figure 2B).^5^

**Figure 3:**
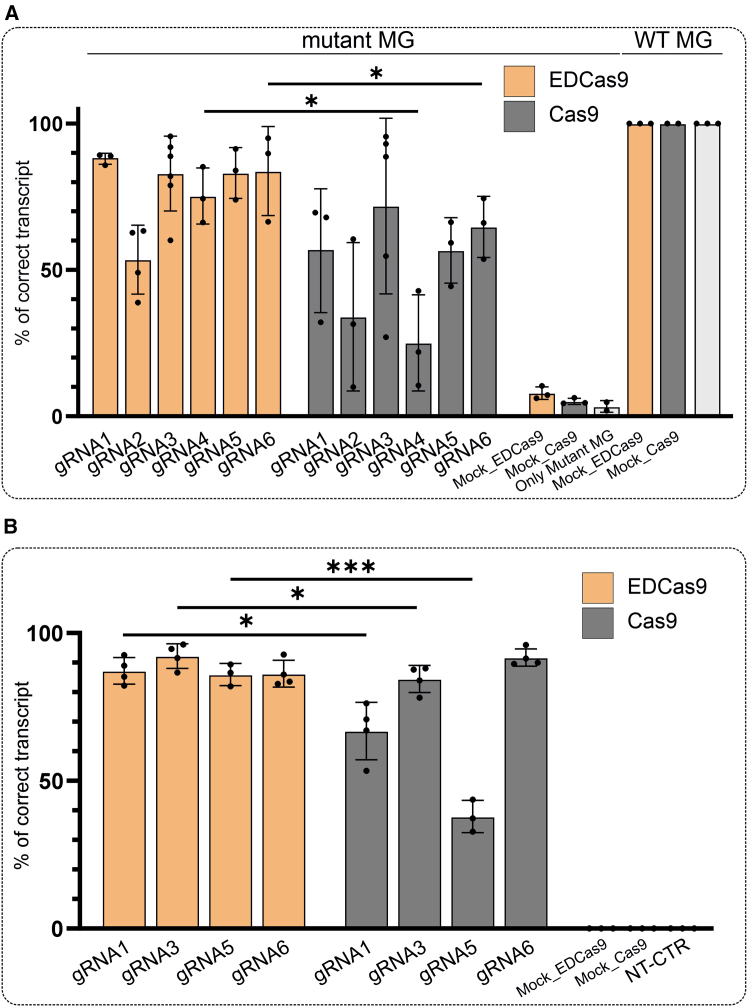
sgRNA/EDCas9 and/Cas9-mediated rescue of the *USH2A*:c.7595-2144A>G-induced splicing defect (A) Minigene splicing correction assay in HEK293T. HEK293T cells were co-transfected with mutant (Mut) or WT *USH2A* minigene (MG) constructs and plasmids encoding for the different sgRNA/EDCas9 or/Cas9 combinations, respectively. The relative proportions (percentages) of correctly spliced transcript as quantified from RT-PCR products are shown. Minigene co-transfection with a plasmid expressing EDCas9 or Cas9 and a scrambled gRNA was used as a mock gRNA control. Transfection of the mutant (NT Mut) and WT (NT WT) minigene plasmids alone was used as a control. (B) Homozygous *USH2A*:c.7595-2144G patient-derived fibroblasts were transfected with plasmids encoding for four lead gRNAs (gRNA1, gRNA3, gRNA5, and gRNA6) alongside EDCas9 or Cas9. Fibroblasts electroporated with a plasmid expressing EDCas9 or Cas9 and a scrambled gRNA were used as a mock gRNA control. Non-transfected (NT-CTR) fibroblasts are also shown in comparison. (A and B) Results are presented as mean ± SD (*n* = 2–6 independent transfections; single data points are shown; Table S1). Statistically significant changes in percentage (%) of correctly spliced *USH2A* transcripts are expressed as ∗*p* ≤ 0.05; ∗∗*p* ≤ 0.01; ∗∗∗*p* ≤ 0.001.

Consequently, HEK293T cells transfected with the mutant and WT plasmids alone resulted in 3.4% ± 2.0% and 100% ± 0% correct *USH2A* transcripts, respectively (Figures 3A and S1A). HEK293T cells co-transfected with the *USH2A*:c.7595-2144A>G mutant minigene and a mock gRNA targeting EDCas9 or Cas9 to a protospacer not found in GRCh38, showed detection of 7.9% ± 2.2% and 5.1% ± 1.0% correctly spliced *USH2A* transcripts, respectively (Table S1). Conversely, co-transfection of the WT minigene and this mock gRNA/Cas9 or gRNA/EDCas9 resulted in a 100% ± 0% correctly spliced *USH2A* transcript.

Next, gRNA/Cas9 and gRNA/EDCas9 combinations were co-transfected along with the mutant *USH2A*:c.7595-2144A>G minigene to evaluate the level of rescued transcript. Overall, all the tested gRNA/EDCas9 combinations exhibited higher splicing rescue than the corresponding gRNA/Cas9 combinations (Figures 3A and S1A; Table S1). Specifically, treatment with the gRNAs and EDCas9 resulted in a range of fractions of correctly spliced *USH2A* transcripts, with gRNA1 exhibiting the highest efficacy at 88.0% ± 1.9% and gRNA2 the lowest at 53.6% ± 11.8%. Conversely, treatment with Cas9 led to lower fractions of correctly spliced *USH2A* transcripts, with gRNA3 achieving 71.8% ± 30.0% and gRNA2 only attaining 34.0% ± 25.3%. In addition to gRNA2/EDCas9, all the other sgRNA/EDCas9 combinations consistently induced high splicing rescue, resulting in a fraction of correctly spliced *USH2A* transcripts greater than 75.3% ± 9.6%. Of note, gRNA/Cas9 combinations produced variable rescue outcomes and exhibited less consistent results across biological replicates (standard deviation [SD] 10.4%–30% for Cas9 vs. 1.9%–15.2% for EDCas9 experiments).

### Lead sgRNA/EDCas9 combinations induce high and consistent splicing rescue in patient-derived homozygous *USH2A*:c.7595-2144G fibroblasts

gRNA1, gRNA3, gRNA5, and gRNA6 had the greatest efficacy in rescuing the splicing defect in minigene assays for both Cas9 and EDCas9 and were chosen for further validation in homozygous *USH2A*:c.7595-2144G patient-derived fibroblasts. Electroporation was used to deliver the genome-editing plasmids into the cells. To limit the variability across electroporation rounds, enhanced green fluorescent protein (EGFP)^+^ cells were sorted 24 h post-electroporation and further cultured until confluent. EGFP was used as a fluorescent marker, expressed in frame with either EDCas9 or Cas9. To prevent possible degradation of the aberrant *USH2A* transcript by NMD, fibroblasts were treated with 0.1 mg/mL cycloheximide, a commonly used NMD blocker, 16 h prior to harvesting.^26^

Mock-transfected fibroblasts show complete aberrant splicing of the *USH2A* transcript, with not even marginal production of the correctly spliced transcript detected (Figures 3B and S1A). Lead gRNA/Cas9 combinations led to different fractions of correctly spliced *USH2A* transcript, ranging from 38.2% ± 5.4% for gRNA5 to 91.8% ± 3.2% for gRNA6, which achieved the highest rescue across the two editing strategies. Conversely, all combinations of lead gRNA/EDCas9 induced ≥85% splicing rescue, ranging from 85.7% ± 3.7% for gRNA5 to 92.4% ± 4.8% for gRNA3 (Figure 3B; Table S1). Notably, the splicing rescue induced by Cas9 differed between the minigene assay and patient-derived fibroblasts, with gRNA5 (56.7% ± 11.2% and 38.2% ± 5.4%, respectively) and gRNA6 (64.7% ± 10.4% and 91.8% ± 3.2%, respectively) showing the greatest variance between the two experimental model systems. In contrast, the splicing correction achieved by EDCas9 was consistent with the results obtained in the minigene assay.

When coupled to EDCas9, gRNA1, gRNA3, and gRNA5 resulted in a higher fraction of correctly spliced transcript compared to the Cas9 combinations, with gRNA1 showing significant differences. Specifically, gRNA1 resulted in 86.6% ± 5.2% and 66.8% ± 8.7% correctly spliced *USH2A* transcripts, gRNA3 in 92.4% ± 4.8% and 84.6% ± 5.0% correctly spliced *USH2A* transcripts, and gRNA5 in 85.7% ± 3.7% and 38.2% ± 5.4% correctly spliced *USH2A* transcripts, respectively. In contrast, gRNA6 coupled to Cas9 exhibited higher splicing rescue compared to EDCas9 (91.8% ± 3.2% and 86.3% ± 4.9%, respectively).

Sequence analysis by amplicon subcloning on residual *USH2A* aberrant transcripts from the genome-edited samples demonstrated the presence of further misspliced transcripts (Figure S1B). In detail, aberrant spliced products resulting in the insertion of shorter PEs were observed in the case of gRNA1/EDCas9, which had lengths of 134 and 136 bp and lacked a portion of the 5′-terminal end sequence of PE40. Additionally, the characterization of the residual misspliced transcripts of gRNA5/EDCas9 showed retention of PEs lacking 3 or 4 bp at the 3′-terminal end, while gRNA5/Cas9 transcripts retained PEs lacking 1, 2, or 4 bp. Notably, gRNA5/Cas9 also retained PEs with single or double nucleotide insertions. In the cases of gRNA1/Cas9 and gRNA6/Cas9, “hybrid” PEs were detected, where part of the retained sequence was derived from another gene, which might point to an off-target site mediating this chromosomal translocation. Specifically, the sequence GenBank: NG_050857.1 (nucleotide position: 85744-85719) was detected for gRNA1/Cas9 and NG_0047091.1:51092–51136 was detected for gRNA6/Cas9.

### EDCas9 consistently induces larger, directional, and localized deletions in PE40-HEK293T and patient-derived fibroblasts

**Figure 4:**
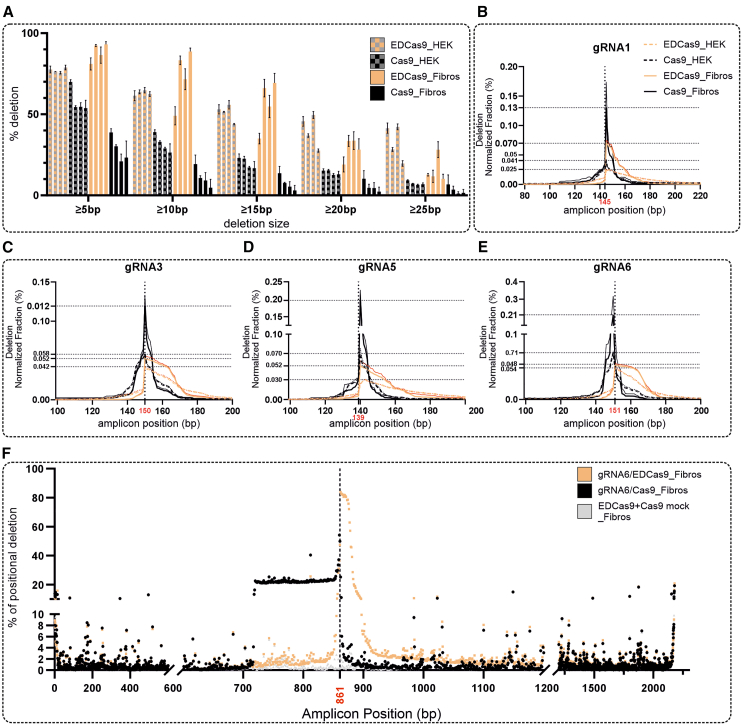
Deletion profiling induced by EDCas9 and Cas9 with lead sgRNAs in PE40-HEK293T and homozygous *USH2A*:c.7595-2144G patient-derived fibroblasts (A) Cumulative frequency (%) of deletions induced by gRNA1, gRNA3, gRNA5, and gRNA6 coupled to EDCas9 or Cas9 detailed for deletions greater than or equal to 5, 10, 15, 20, and 25 bp . (B–E) Deletion profiling for the lead gRNA1 (B), gRNA3 (C), gRNA5 (D) and gRNA6 (E). The y axis represents the normalized deletion frequency (%) at each position of the sequenced amplicon (x axis). The dashed line indicated the Cas9 cleavage site. The data are represented as mean ± SD (*n* = 2–3 independent experiments). (F) Fraction (%) of positional deletion at each position of the 2,178-bp amplicon normalized to the position having the highest coverage. The amplicon was amplified from fibroblasts treated with gRNA6/EDCas9 and/Cas9 and sequenced by nanopore long-read sequencing. The data are represented as a single experiment where amplicons from two independent samples were equimolarly pooled and sequenced. Note that individual data points with apparent high percentages of positional deletions represent technical nanopore sequencing errors (e.g., homo-oligomeric sequences).

To quantify and profile the genomic DNA cleavage outcomes of EDCas9 and Cas9 in conjunction with lead gRNAs, a stable polyclonal cell line (PE40-HEK293T) was generated via lentiviral transduction of a transgene containing a genomic region of 425 bp of *USH2A* intron 40, including the c.7595-2144G pathogenic variant. Upon transfection with gRNA/EDCas9 or gRNA/Cas9 expression constructs, transgene-specific high-throughput sequencing (HTS) was conducted to assess the resulting editing profiles. Genomic DNA of the gRNA/(ED)Cas9- and /Cas9-treated patient-derived fibroblasts was also subjected to HTS and the results compared to those obtained in PE40-HEK293T. The analysis of the data showed similar genomic DNA cleavage efficacy across the tested gRNAs and between EDCas9 and Cas9 in both cellular models, as defined by the percentage of mutational repair profiles (i.e., indels) (Figure S2). However, the type of the resulting deletion profiles varied substantially. While Cas9 predominantly resulted in the deletion of a few nucleotides, EDCas9 induced consistent and directional deletion of larger sequence stretches (Figures 4A–4E). For the four lead gRNAs, EDCas9 induced deletions of at least 5, 10, and 20 bp at average rates of 77% ± 1%, 63% ± 1%, and 40% ± 10% in PE40-HEK293T cells, and 88% ± 6%, 73% ± 18%, and 29% ± 7% in *USH2A*:c.7595-2144G patient-derived fibroblasts, respectively. In contrast, Cas9 resulted in lower deletion rates: 58% ± 8%, 32% ± 5%, and 14% ± 1% in PE40-HEK293T cells, and 28% ± 8%, 11% ± 6%, and 5% ± 3% in patient-derived fibroblasts (Figure 4A). This suggested less variability in the deletion spectrum induced by EDCas9 across the two cellular models compared to Cas9. Subsequent direct comparison of deletion profiles, normalized to account for insertion and substitution events and differing cleavage efficiencies, also revealed greater variability in the fraction of Cas9-induced events compared to EDCas9-induced events (Figures 4B–4E). Notably, despite the higher variability in fraction compositions, the types of mutational profiles induced by Cas9 were mostly consistent across both cellular models, as confirmed by CRISPResso analysis (Figure S3A). Furthermore, the direction of EDCas9-induced deletions was consistently oriented toward the 5′ end of the non-target strand (Figures 4B–4E). With respect to insertion events at the cleavage site, the use of EDCas9 drastically reduced the occurrence of insertions in comparison to Cas9 (Figure S3B).

To more comprehensively assess the extension of the deletions generated by gRNA6/EDCas9 in comparison to gRNA6/Cas9 and EDCas9 or Cas9 mock in *USH2A*:c.7595-2144G patient-derived fibroblasts, a larger amplicon of 2,178 bp was sequenced by nanopore technology (Figures 4F and S4A–S4C). Alignments of the nanopore reads to the amplicon reference sequence confirmed the presence of deletions for both gRNA6/EDCas9- and gRNA6/Cas9-treated fibroblasts, while, as expected, they were absent in EDCas9 and Cas9 mock-treated cells. Interestingly, gRNA6/Cas9 fibroblasts showed a predominant deletion of ∼140 bp. This deletion spans the whole PE sequence, including the *USH2A*:c.7595-2144G pathogenic variant. For gRNA/EDCas9, the resulting deletion profile confirmed the presence of larger deletions, with the vast majority directionally extending ∼100 bp upstream to the cleavage site on the targeted strand. A few deletions extend further, up to ∼400 bp. Beyond that size, there was no difference compared to background (i.e., EDCas9 or Cas9 mock samples).

### Safe off-target profiles of both lead gRNA3 and gRNA6 conjugated to either EDCas9 or Cas9, with gRNA6/EDCas9 shown to prevent chromosomal translocations

**Figure 5:**
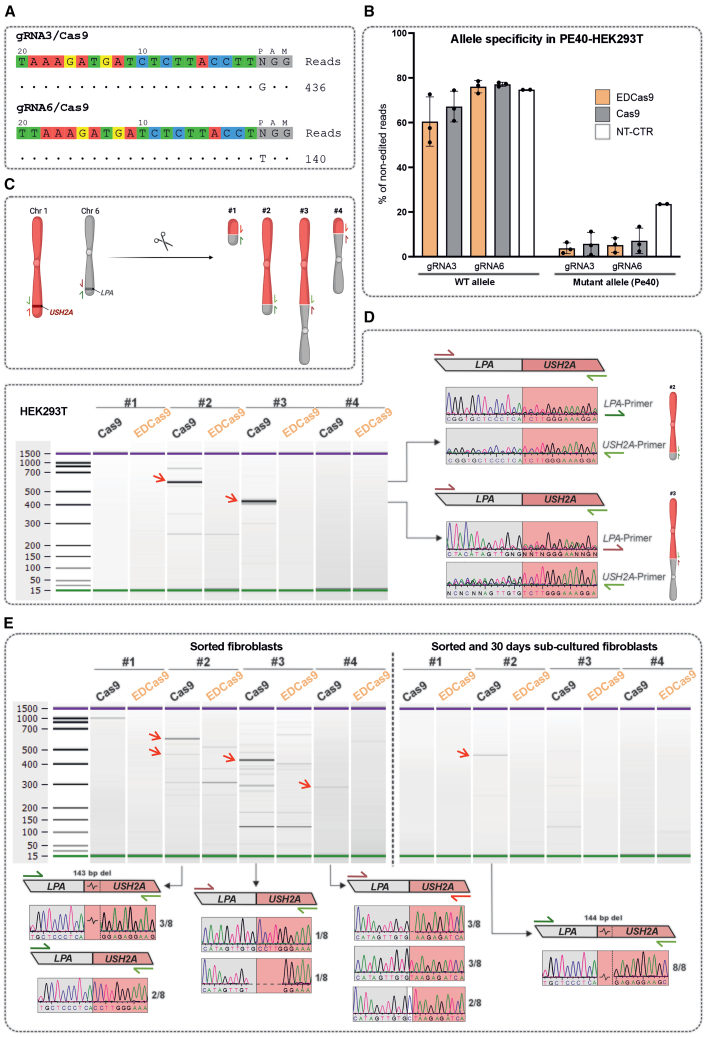
Off-target assessment of lead gRNA3 and gRNA6, and targeted translocation assay of lead gRNA6 (A) GUIDE-seq assay using Cas9 with gRNA3 or gRNA6 in PE40-HEK293T cells revealed no nominated off-target sites. (B) Allele-specificity analysis of gRNA3 or gRNA6 coupled to Cas9 or EDCas9, respectively, in PE40-HEK293T cells measured as percentage (%) of non-edited reads in HTS amplicon sequencing. Non-transfected cells (NT-CTR) served as a control. Data are presented as mean ± SD from *n* = 2–3 independent experiments. (C) Schematic representation of the targeted translocation assay. The *USH2A* locus on chromosome 1 and the *LPA* locus on chromosome 6 were simultaneously targeted and potentially result in four distinct translocation outcomes. Red and green arrows indicate forward and reverse primers used for detecting translocated chromosomes. (D) Translocation assay in HEK293T cells, showing translocation products visualized via electrophoresis of PCR products. Gel-purified bands were validated by Sanger sequencing, confirming the identity of the amplicons. Red arrows mark the purified bands. All the EDCas9 and Cas9 samples were processed following the same workflow. (E) Translocation assay in homozygous *USH2A*:c.7595-2144G patient-derived fibroblast cells, demonstrating translocation-derived PCR products on an electrophoresis gel. Validation was performed by subcloning and Sanger sequencing, confirming the amplicons’ identity. Red arrows indicate bands corresponding to characterized translocation outcomes based on size. Adjacent to each Sanger sequencing trace, the observed frequency of single-clone sequencing results is reported. All the EDCas9 and Cas9 samples were processed following the same workflow.

To address the off-target potential of the leads gRNA3 and gRNA6, a combination of unbiased genome-wide, unbiased identification of DSBs enabled by sequencing (GUIDE-seq) and targeted HTS was used. As the targeting specificity of the two nucleases is determined by the nuclease activity, GUIDE-seq was performed for Cas9. The assay was done in PE40-HEK293T cells, which harbors the gRNA on-target sequence used as a positive control for the workflow. For both gRNA3 and gRNA6, GUIDE-seq nominated no off-target sequence, while showing successful nomination of the on-target site (Figure 5A). In addition, the three most likely predicted off-target sites based on mismatches to the gRNA sequence were determined and tested. Validation of these showed the absence of editing above the 0.1% threshold as determined by the CRISPECTOR tool (Figure S5A).

Allele specificity for gRNA3 and gRNA6 was also determined in PE40-HEK293T by using primers for PCR amplifications that amplify both the PE40 transgene and *USH2A* endogenous WT region. The HTS results from the non-transfected cells indicate a 1:3 ratio of PE40 transgene to *USH2A* endogenous locus, respectively. As determined by the fraction of non-edited reads for the PE transgene (*USH2A*:c.7595-2144G) compared to the endogenous *USH2A* sequence (*USH2A*:c.7595-2144A), both gRNAs, regardless of conjugation with EDCas9 or Cas9, showed a clear targeting preference for the mutant allele, with gRNA6 showing higher specificity (Figure 5B).

Furthermore, we sought to determine whether EDCas9 conjugating with the lead gRNA6 targeting *USH2A* PE40 would also reduce the formation of targeted chromosomal translocations as reported previously for another Cas9, the TREX2 fusion system.^23^^,^^24^ To test this, HEK293T and patient-derived fibroblasts were co-transfected with gRNA6(WT) and gRNA-Lipoprotein(a) (LPA) or gRNA6 and gRNA-LPA, respectively, conjugated to EDCas9 or Cas9. The additional gRNA-LPA is a highly active gRNA targeting *LPA*, which mimics a potential off-target or random DSB on chromosome 6. Translocation between the two target sites could lead to four different translocation outcomes (1–4) that can be specifically amplified by PCR (Figure 5C). From treated HEK293T cells, genomic DNA was extracted from the entire pool of cultured cells 72 h post-transfection. To account for the variability across electroporation rounds observed in fibroblasts, cells were flow-sorted for (ED)Cas9-EGFP^+^ cells 72 h post-treatment. Sorted cells were used directly for DNA extraction or re-seeded and cultured for another 30 days—thereby assessing a potential loss of chromothripsis-damaged cells—before DNA extraction. In HEK293T cells, gRNA6/EDCas9 resulted in the absence of translocations to the level of detection. In contrast, gRNA6/Cas9-treated cells yielded to a strong amplification for translocation events 2 and 3, which were confirmed by sequencing (Figures 5D, S5B, and S6). In fibroblasts treated with gRNA6/Cas9, sorted and processed after 72 h, discrete amplification products were observed with primer sets for events 2, 3, and 4, matching the expected sizes of the fusion products. However, additional bands were also detected in samples treated with gRNA6/EDCas9 or gRNA6/Cas9. To determine the identity of these bands, gel purification, subcloning, and sequencing of individual colonies was performed. This analysis confirmed the presence of translocations exclusively in the gRNA6/Cas9 samples. In contrast, for gRNA6/EDCas9, the subcloned fragments’ sequence could not be uniquely mapped to any specific human sequence, suggesting the possibility of non-specific PCR products (data not shown). Finally, patient-derived fibroblasts that were sorted and sub-cultured for 30 days showed the persistent presence of a product for primer set 2 in gRNA6/Cas9-treated cells. Notably, the detected chromosomal translocation was characterized to include a 144-bp deletion mediated by gRNA6 (Figures 5E and S5B).

Since nanopore sequencing revealed the presence of a large ∼140-bp deletion associated with gRNA6/Cas9 at the *USH2A* locus in fibroblasts, for this experiment, on-target cleavage activity was performed using primers annealing outside the deleted region. Notably, on-target editing efficacy at the *USH2A* cleavage site was similar for EDCas9 and Cas9, while at the *LPA* cleavage site, EDCas9 yieldedto higher editing compared to Cas9. However, the genomic deletion of ∼140 bp induced by gRNA6/Cas9 at the *USH2A* cleavage site (observed in the sgRNA experiments) was again consistently observed only in fibroblasts but absent in HEK293T cells (Figures S6–S8).

### Virus-like particles enable delivery of gRNA6/EDCas9

**Figure 6:**
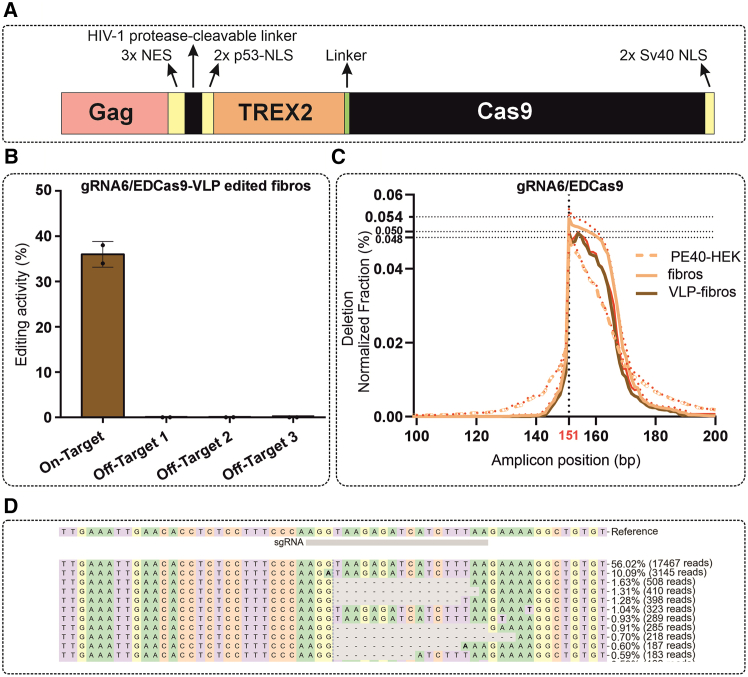
gRNA6/EDCas9 delivery mediated by virus-like particles in homozygous *USH2A*:c.7595-2144G patient-derived fibroblasts (A) Schematic representation of the EDCas9 design used in the experiments for VLP production. NES, nuclear export signal; NLS, nuclear localization signal. (B) Editing activity upon VLP-mediated delivery of gRNA6/EDCas9 of the on-target site and the three predicted off-target sites. Results are presented as mean ± SD (*n* = 2 independent transduction experiments). (C) Deletion profiling for gRNA6/EDCas9 plasmid construct lipofected into PE40-HEK293T or electroporated into patient-derived fibroblasts (Fibros), and gRNA6/EDCas9 RNPs delivered by VLPs into patient-derived fibroblasts (VLP-Fibros) The y axis represents the normalized deletion frequency (%) at each position of the sequenced amplicon (x axis). The dashed line indicates the Cas9 cleavage site. The data are represented as mean ± SD (*n* = 2–3 independent experiments). (D) CRISPResso allele frequency table showing presence of the WT *USH2A* sequence (c.7595-2144A, second line, 10.09%, 3,145 reads).

To further evaluate the therapeutic potential of gRNA6/EDCas9, we investigated its delivery via virus-like particles (VLPs). The VLP design described by Hamilton and co-workers was adapted to the delivery of EDCas9 (Figure 6A).^27^ Successful generation of VLPs containing EDCas9 ribonucleoproteins (RNPs) was confirmed by dynamic light scattering (DLS), showing a homogeneous particle size distribution of 159 ± 4 nM, and protein dot blot analysis, detecting packed Cas9 protein (Figures S9A and S9B). The ability of the VLP-delivered gRNA6/EDCas9 RNPs to achieve editing was tested in patient-derived fibroblasts. For this purpose, 1 pmol (equivalent to 2.5 × 10^8^ particles) of gRNA6/EDCas9 packaged VLPs was applied to ∼20,000 fibroblast cells. Seventy-two hours post-treatment, DNA cleavage efficacy, deletion profile formation, and off-target activity were tested. The results confirmed the editing activity of the gRNA6/EDCas9 RNP when delivered as VLPs, achieving an indel formation efficiency of 36% ± 3% (Figure 6B). Importantly, the deletion profiles closely mirrored those observed upon delivery of plasmid constructs into PE40-HEK293T cells and patient-derived fibroblasts via lipofection or electroporation, respectively (Figure 6C). Furthermore, analysis of predicted off-target sites revealed no detectable editing activity at these sequences (Figure 6A).

Interestingly, HTS revealed the presence of WT reads in the VLP-treated samples but not in the non-treated sample (Figures 6D and S9C), indicating the carryover of DNA material from the producer cells (HEK293T). This observation was further validated using gene-specific PCR for *USH2A* and *RHO*, which confirmed amplification in the purified VLP preparations (Figure S9D). However, the complete absence of edited reads traceable to the WT allele excludes the possibility of editing bias arising from editing events in the producer cells during VLP production.

## Discussion

Due to their location remote from coding sequences, increasing therapeutic interest is placed on addressing pathogenic deep-intronic variants affecting splicing. In this respect, ASOs have emerged as effective molecules for splicing correction.^5^^,^^8^^,^^9^^,^^11^ However, their action at the pre-mRNA level results in a temporal effect that does not tackle the underlying genetic cause, rendering recurrent administration necessary. In cases of ocular or retinal disease, this goes along with recurrent intravitreal injections with risks of bleeding, cataracts, and intraocular infection.^11^^,^^28^^,^^29^^,^^30^ In contrast, by targeting the genomic level, genome-editing technologies provide a promising avenue to permanently address splicing defects induced by pathogenic deep-intronic variants.^12^^,^^13^^,^^14^^,^^15^^,^^31^ However, current gene-editing approaches aiming to rescue splicing are typically tailored to result in the deletion of a genomic fragment encompassing the PE sequence, which is mediated by two gRNAs. While effective, these editing designs raise concerns about potential chromosomal instability due to the introduction of multiple DSBs and the generation of a fragmental sequence deletion.^12^^,^^14^^,^^18^^,^^31^ Therefore, an sgRNA approach is favored, which relies on the perturbation of crucial (mis)splicing sequence elements (e.g., cryptic splice sites) through editing events induced by non-homologous end joining (NHEJ), which in turn prevents the spliceosome from their recognition, leading to the restoration of correct splicing.^14^^,^^15^

In an attempt to treat the splicing defect caused by the recurrent *USH2A*:c.7595-2144A>G deep-intronic variant, we explored the use of Cas9 and EDCas9, only employing sgRNAs. Given that WT Cas9 coupled to a sgRNA typically induces limited sequence perturbation by creating small indels, we hypothesized that using the Cas9-TREX2 fusion protein (EDCas9), which reportedly increases the size and frequency of deletions, could be more effective in achieving splicing rescue.^21^ Indeed, all six sgRNAs tested in the minigene assay demonstrated higher splicing correction when paired to EDCas9 in comparison to Cas9. Subsequent validation of four lead gRNAs (gRNA1, -3, -5, and -6) coupled with EDCas9 in patient-derived fibroblasts affirmed robust splicing rescue (>85%), with results in line with those obtained in minigene experiments. Notably, variability and differences emerged for Cas9, highlighting the potential impact of diverse mutational profiles in different cell lines on the outcomes of splicing rescue.^21^^,^^32^ This was particular evident for gRNA5, inducing 56.7% ± 11.2% vs. 38.2% ± 5.4% of correctly spliced transcript and gRNA6, resulting in 64.7% ± 10.4% vs. 91.8% ± 3.2%, in minigene assay and patient-derived fibroblasts, respectively. Furthermore, in patient-derived fibroblasts, only two gRNAs paired to Cas9 (gRNA3 and gRNA6) led to a considerable fraction of correct transcript >80%, limiting the number of effective gRNAs to two as opposed to four for EDCas9.

Further analysis of mutational outcomes at the genomic level revealed significant differences in the fraction of deletions between the two cellular models when using Cas9. These differences likely contribute to the observed variability in splicing rescue (i.e., as an outcome of a productive editing event) for certain gRNAs coupled with Cas9. In contrast, the activity of TREX2 in EDCas9 influences the repair outcome, yielding more consistent editing profiles across the two cellular models and under different delivery modalities. This consistency is advantageous for designing preclinical *in vitro* experiments that aim to generate translatable data for therapeutic development. Further exploration of this phenomenon across diverse cell types and advanced *in vitro* tissue models will provide deeper insights into its mechanisms and enhance its applicability for clinical translation.

It is also crucial to highlight that the higher splicing rescue achieved with EDCas9 was not attributed to a higher cut efficiency at the genomic level, as it is comparable to that achieved with Cas9. When analyzing the resulting genomic mutational profiles, it is evident that EDCas9 boosts the occurrence of deletions as well as the size of these deletions, while drastically reducing the manifestation of insertions. More specifically, the cleavage kinetic of EDCas9 induces highly directional deletions biased toward the 3′ end of the non-target strand. This occurrence is attributed to the preferential dissociation of Cas9 from the 3′end of the non-target strand during cleavage, while maintaining stable engagement with the remaining three ends of the DSB. In this scenario, the released end (3′ end of the non-target strand) is immediately accessible for processing by TREX2, thereby resulting in deletions biased toward this specific direction.^25^

To further investigate whether the deletion outcomes achieved by gRNA6/EDCas9 were localized or extended beyond the short amplicon analyzed through HTS, we amplified a larger genomic fragment of 2,178 bp around the targeted *USH2A* site in treated patient-derived fibroblasts and analyzed it using nanopore sequencing. Results confirmed that gRNA6/EDCas9 introduced directional and localized deletions in a range of <50 bp for ∼95% of the reads (from position 861–910 bp in Figure 4F).^23^ In contrast, gRNA6/Cas9 yielded a major deletion of ∼140 bp, which was not initially detected in HTS experiments as it is located outside the short amplicon used. The presence of this deletion in fibroblasts was further validated by HTS in independent transfection experiments. Interestingly, this deletion outcome was absent at the endogenous locus in HEK293T cells, providing additional evidence of the variability in Cas9-generated mutational profiles across different cell lines.

Off-target evaluation using the unbiased GUIDE-seq protocol and HTS of three predicted off-target sites for gRNA3 and gRNA6 revealed high specificity for the target sites, with no GUIDE-seq-nominated off-targets or detectable editing activity at the tested loci for both Cas9 and EDCas9. For gRNA6/EDCas9, the absence of editing at predicted off-target sites was also confirmed when delivered via VLPs. Despite targeting an intronic sequence, allele-specific editing is a highly desirable feature in the design of gene-editing strategies. Allele-specificity experiments in PE40-HEK demonstrated high allele specificity for gRNA6 when combined with either EDCas9 or Cas9. gRNA3 showed less allele specificity and was more pronounced with EDCas9. However, this trend must be considered alongside the increased editing observed at the mutant *USH2A* allele for gRNA3/EDCas9.

Additionally, the application of EDCas9 reduces the risk of chromosomal translocations as detected with standard Cas9 in the two tested cellular models. Although we showed that gRNA6 is highly specific for the target with no detectable off-target event and thus per se reduced the risk for chromosomal translocations due to multiple DSBs for this very gRNA, the absence of targeted chromosomal translocation for EDCas9 in the two cellular models described an additional safety feature of the editing strategy. Notably, Cas9-mediated re-cutting events due to the scarless repair of the target sequence by NHEJ can lead to other target aberrations, such as loss of heterozygosity, or it can result in higher P53-mediated cytotoxicity due to recurrent generation of DSBs over time.^18^^,^^33^^,^^34^^,^^35^^,^^36^^,^^37^

Since EDCas9 has been reported to reduce the occurrence of chromosomal translocations by preventing NHEJ-mediated indel-free rejoining of the target sequence, thereby abolishing Cas9-mediated re-cutting events, we investigated whether this also applied to the lead gRNA6.^23^

When coupled with Cas9, gRNA6 resulted in detectable targeted chromosomal translocations in both HEK293T and patient-derived fibroblast cells. Interestingly, the translocation involving the telomeric segment of chromosome 6, where *LPA* is located, and the long arm of chromosome 2, where *USH2A* resides, persisted even -after 30 days of sub-culturing. This suggests that some translocated chromosome remained stable and can successfully segregate. Notably, only the translocated chromosome carrying the 143-bp deletion in *USH2A* was detected.

A crucial aspect of EDCas9 in correcting splicing due to PE retention lies in the use of the human TREX2 exonuclease. It is important to consider that, by definition, TREX2 may possess the capacity to process not only the intended target sequence specified by the gRNA/Cas9 complex but also other free DNA ends, potentially raising safety concerns. However, studies have demonstrated that the inactivation of TREX2’s DNA-binding domain through targeted mutagenesis preserves the catalytic ability of the Cas9-exo fusion endonuclease while eliminating its capacity to bind DNA.^22^^,^^23^^,^^24^^,^^38^ This provides an engineering solution to mitigate safety concerns that might be linked to the proposed editing approach, which will be explored in future experiments.

In inherited retinal disease, the gold standard delivery method for gene therapy is the AAV.^39^ AAV-mediated delivery provides long-term expression of the transgene, a highly favorable characteristic for gene-augmentation approaches. However, for gene-editing applications, the transient expression of the editing molecule is preferred to minimize off-target effects and other complications associated with prolonged activity.^40^^,^^41^^,^^42^ To address this limitation, VLPs have emerged as a promising alternative. VLPs are HIV-based particles loaded with proteins rather than genomic material, resulting in their ability to deliver gene-editing components transiently. Notably, VLPs have demonstrated efficacy in mediating gene editing in the retinal pigment epithelium in the mouse retina, highlighting their potential for ocular gene therapy applications.^43^^,^^44^ Building on this foundation, we explored whether VLPs could also mediate the delivery of EDCas9. Our experiments demonstrated the viability of VLP-mediated EDCas9 delivery, confirming that the system is capable of delivering functional EDCas9 and achieving the expected mutational profiles characterized by larger and directional deletions.

In conclusion, we applied two sgRNA CRISPR-Cas9 approaches to target the recurrent *USH2A*:c.7595-2144A>G deep-intronic variant, achieving effective splicing restoration in both minigene assays and patient-derived fibroblasts. While standard Cas9 editing exhibited inconsistent splicing rescue and divergent mutational profiles between these models, suggesting challenges in translating finding of sgRNA/Cas9 approaches across different cellular contexts, EDCas9 achieved similar repair mutational profiles and consistent splicing correction in both systems, positioning it as a promising tool for splicing correction. Additionally, VLP-mediated EDCas9 delivery demonstrates potential as a therapeutic platform for addressing pathogenic variants that benefit from larger, directional deletions, such as those involved in splicing modulation or the removal of regulatory elements.

## Materials and methods

### gRNA design

Suitable gRNAs were designed on Benchling.com.

### Plasmids

The mutant (*USH2A*:c.7595-2144G) and the WT (*USH2A*:c.7595-2144A) minigene plasmids were generated by Q5 High-Fidelity DNA Polymerase (New England Biolabs [NEB], catalog no. M0491) amplification of the target *USH2A* region using gDNA of a human heterozygous *USH2A*:c.7595-2144A/G patient and subsequent cloning into the pSPL3 backbone vector by NEBuilder HiFi DNA Assembly Cloning Kit (NEB, catalog no. E5520S). The intronic sequence encompassing the *USH2A*:c.7595-2144 location was introduced in the recipient pSPL3 exon trapping vector, including the intronic sequence 966 bp upstream and 914 bp downstream of the *USH2A*:c.7595-2144 location. Primers are listed in Table S2.

The standard *Cas9* plasmid is represented by PX458 (Addgene, catalog no. 48138). To generate the EDCas9 (3xFLAG-SV40 NLS-TREX2-linker-SpCas9) plasmid, the 3xFLAG-SV40 NLS-TREX2 sequence was cloned at the N terminus of *Cas9* by the NEBuilder HiFi DNA Assembly Cloning Kit. Specifically, the PX458 vector was digested by *Age*I and *Eco*RV, the 3xFLAG-SV40 NLS was amplified from the PX458 vector, the TREX2-linker fragment was amplified from the pKLV2.2-TREX2-linker-Cas9 plasmid (generously provided by Dr. Andrew Bassett, Sanger Institute, Hinxton, UK), and the part of the Cas9 sequence digested was amplified back from PX458.

The transfer PE40-USH2A lentivirus plasmid was cloned by digesting the pKLV2.2-mU6gRNA5(SapI)-hU6gRNA5(BbsI)-PGKpuroBFP-W (Addgene, catalog no. 72667) lentiviral transfer plasmid by *Apa*I and *Bam*HI. The PE40 fragment was amplified from patient genomic DNA using overhanging primers. The backbone plasmid and fragment were cloned using the NEBuilder HiFi DNA Assembly.

The Gag-TREX2-SpCas9 VLP plasmid was cloned by digesting the pJRH-1179 U6-reci Gag-Cas9 v2 plasmid (Addgene, catalog no. 201914) by *Age*I and *Eco*RV. The excised 2xP53NLS fragment was amplified back from the same plasmid. The fragment containing *TREX2* and part of *Cas9* were amplified from the EDCas9 plasmid.

High-Fidelity DNA Polymerase (NEB) was used for fragment amplification. The fragments and backbone plasmids were cloned following the manufacturer’s protocol. The primers are listed in Table S2.

The Zhang lab general cloning protocol was used to insert annealed synthetic-oligonucleotide gRNA into the *Bbs*I restriction site to clone sgRNAs into PX458 and EDCas9.^45^ The gRNA sequences are listed in Table S2. Plasmid sequences are provided in Table S3.

### Cell lines and culture conditions

HEK293T cells (American Type Culture Collection, 293T/17) were cultured in Dulbecco’s modified Eagle’s medium (DMEM; Thermo Fisher Scientific, catalog no. 41966029) supplemented with 10% fetal bovine serum (FBS; Thermo Fisher Scientific, catalog no. 10270106), 10 U/mL penicillin/streptomycin (Thermo Fisher Scientific, catalog no. 15140122) at 37°C in a 5% CO_2_ humidified atmosphere. PE40-HEK293T cells were maintained as described for HEK293T but cultured in DMEM supplemented with 10% FBS, 10 U/mL penicillin/streptomycin, and 3 μg/mL puromycin (Thermo Fisher Scientific, catalog no. A1113803).

A skin biopsy of a *USH2A* patient was obtained upon informed written consent, complying with the guidelines and approved by the local ethics committee (project no. 124/2015BO1). Patient-derived fibroblast cells were expanded and cultured in DMEM supplemented with 20% FBS and 10 U/mL penicillin/streptomycin at 37°C in a 5% CO_2_ humidified atmosphere. The genotype of the patient-derived cell line is *USH2A*:c.[7595-214A>G]; [7595-214A>G] (further denoted as fibroblasts).

### Transfection of cell lines

HEK293T cells were seeded in a 24-well plate (250,000 cells/well) in DMEM without penicillin/streptomycin, followed by overnight incubation at 37°C in a 5% CO_2_ humidified atmosphere. Cells were transfected using Lipofectamine 3000 (Thermo Fisher Scientific) with 500 ng total plasmids (copy ratio for minigene assay 1:10, minigene: Cas9 or EDCas9 plasmid). After 24 h, the cell medium was changed, and cells were harvested 48 h post-transfection for mRNA isolation.

Patient-derived fibroblasts were transfected using the Neon electroporation system, according to the manufacturer’s instructions (Thermo Fisher Scientific, catalog no. MPK5000). Briefly, cells were detached by trypsin-EDTA (0.05%) (Thermo Fisher Scientific, catalog no. 25300054) (5 min at 37°C), harvested in 10 mL DMEM, and collected by centrifugation at 300 × *g* for 6 min. To prepare for electroporation, 500,000 cells/reaction were resuspended in 100 μL Buffer R and 5 μg endotoxin-free plasmid(s) (editing plasmid:cells ∼250,000:1) was used per electroporation reaction. Endotoxin-free plasmids were prepared using the EndoFree Plasmid Maxi Kit (Qiagen, catalog no. 12362) following the manufacturer’s protocol. Electroporation was performed at 1,400 V, 20 ms, and 2 pulses. Electroporated cells were immediately plated in a well of a 6-well plate with 2 mL DMEM without penicillin/streptomycin.

### Fluorescence-activated cell sorting

After 24 h, electroporated fibroblasts were sorted for EGFP^+^ cells. Cells were washed in PBS and detached by trypsin-EDTA (0.05%, 5 min at 37°C), harvested in 10 mL DMEM, and collected by centrifugation at 300 × *g* for 6 min. The cell pellet was resuspended in 300 μL PBS. Cell sorting was performed on an MA900 Multi-Application Cell Sorter (Sony Biotechnology). The cells were first gated for forward and side scattering, and then EGFP intensity was measured by a 488-nm blue laser. The maximal amount of cells was sorted (5,000–50,000 cells) and plated back until confluence was reached.

### Lentivirus production and transduction

Lentiviral particles were produced in HEK293T cells at 80% confluence in 10-cm plates transfected by Lipofectamine 3000 according to the manufacturer’s protocol. A second-generation approach was used, employing pMD2.G (Addgene, catalog no. 12259), psPAX2 (Addgene, catalog no. 12260), and transfer vector pKLV2.2-PE40-USH2A. The lentiviral supernatant was harvested at 72 h post-transfection, filtered through a 0.45-μm pore size filter. Lentiviral particles were stored at −80°C.

For the generation of PE40-HEK293T, 100,000 cells were seeded in a 24-well plate in complete DMEM medium and 8 μg polybrene (Merck, catalog no. TR-1003-G), and 10 μL of filtered lentivirus supernatant was added to the cells. After 72 h, the medium was replaced with complete DMEM and 3 μg/mL puromycin to start selection.

### EDCas9 VLPs

EDCas9 VLPs were produced as described by Hamilton et al. ^27^ In brief, three 10-cm dishes were transfected with pMD2.G (Addgene, catalog no. 12259), psPAX2 (Addgene, catalog no. 12260), and the Gag-TREX2-Cas9 plasmid using Lipofectamine 3000. After 72 h, the supernatant was harvested and filtered through a 0.45-μm filter. VLPs were concentrated by ultracentrifugation in a 20% sucrose cushion at 100,000 × *g* for 2 h at 4°C. The VLP pellet was resuspended in Opti-MEM (Thermo Fisher Scientific, catalog no. 31985070). Particle counting and size distribution were performed on a Zetasizer Advance (Malvern Panalytical). VLPs were titrated for Cas9 protein content using dot blot. Five microliters of concentrated VLP were resuspended in 2× dye-free Laemmli buffer and 2 μL spotted on a nitrocellulose membrane. Recombinant Cas9 (NEB, catalog no. M0386T) was used to establish the standard curve. Cas9 mouse monoclonal antibody (Cell Signaling Technology, catalog no. 14697) 1:1,000 diluted was used for staining and conjugated to horseradish peroxidase for bioluminescence detection. Acquired pictures were processed on ImageJ for protein quantification.

### Splicing analysis

Total RNA of minigene-transfected HEK293T cells and patient-derived fibroblasts were extracted using the peqGOLD Total RNA Kit (VWR Life Science, catalog no. 12-6834-02). One microgram of RNA was treated with 1 U DNaseI (Sigma-Aldrich, catalog no. AMPD1-1KT) following the manufacturer’s instructions for 15 min at room temperature, followed by a 10-min heat inactivation step at 70°C after addition of 1 μL stop buffer. DNaseI-treated RNA samples were used for cDNA synthesis. The Maxima H Minus First Strand cDNA Synthesis (Thermo Fisher Scientific, catalog no. K1652) was used for HEK293T-derived samples and the SuperScript IV First-Strand Synthesis System (Thermo Fisher Scientific, catalog no. 18091050) was used for fibroblast-derived samples. A plasmid-specific primer (pSPL3_SA2_R) was used for cDNA synthesis of HEK293T-derived samples, while random hexamers were used in the fibroblast-derived samples. In both cases, the manufacturer’s protocol was followed.

Two microliters of the cDNA were used for PCR amplification employing Taq polymerase (Genaxxon Bioscience, catalog no. M3001.0250) for HEK293T-derived samples and Q5 High-Fidelity DNA Polymerase. The primers are listed in Table S2. PCR products were purified using AMPure XP beads (Beckman Coulter, catalog no. A63880) as per the manufacturer’s protocol. Purified samples were analyzed on a 2100 Bioanalyzer instrument employing DNA 1000 Kit reagents (Agilent Technologies, catalog no. 5067-1504) according to the manufacturer’s protocol. The percentage of correctly spliced transcripts was calculated using the formula (CP/[CP + AP]) × 100, where CP and AP are the molarity of the fragment corresponding to the correctly spliced RT-PCR product and aberrantly spliced RT-PCR product(s), respectively.

### GUIDE-seq

PE40-HEK293T cells were transfected with 250 ng editing plasmid and 5 pmol double-stranded oligo donor using jetOPTIMUS (Polyplus, catalog no. 101000051). Four days post-transfection, genomic DNA was isolated with the DNA Mini Kit (Qiagen, catalog no. 51304) according to the manufacturer’s protocol. HTS library preparation was performed as described by Tsai et al. and sequenced by Novogene.^46^ Fastq files were analyzed using the pipeline provided by the Tsai lab in Github (https://github.com/tsailabSJ/guideseq).

### Chromosomal translocation assay

Cells were pelleted and the genomic DNA was extracted using QuickExtract DNA Extraction Solution (Lucigen, catalog no. 101098) following the manufacturer’s protocol. The amount of genomic DNA was quantified using Qubit. PCR amplification was performed using Q5 High-Fidelity DNA Polymerase. The resulting amplicons were resolved by automated electrophoresis on a 2100 Bioanalyzer System employing DNA 1000 Kit reagents. The primers are listed in Table S2.

### Sanger sequencing

For sequencing PCR amplification products resulting in multiple bands, the PCR products were cloned by CloneJET PCR Cloning Kit (Thermo Fisher Scientific, catalog no. K1232) following the manufacturer’s protocol. Plasmid constructs were verified by Sanger sequencing using the sequencing primers listed in Table S2. Plasmid DNA was extracted from bacterial cultures using the Monarch Plasmid Miniprep Kit (NEB, catalog no. T1010) and sequenced using the BigDye Terminator version 1.1 kit (Thermo Fisher Scientific, catalog no. 4337450) according to the manufacturer’s protocol. The same sequencing protocol was used to verify the success of gRNA cloning in the different backbone vectors. Sequencing of PCR amplicons resulting in a single band was carried out using the BigDye Terminator version 1.1 kit according to the manufacturer’s protocol. Sequencing reactions were resolved on an ABI PRISM 3130xl Genetic Analyzer.

### Long-read nanopore sequencing

The *USH2A* genomic fragment to be sequenced was amplified by Q5 High-Fidelity DNA Polymerase. The primers are listed in Table S2. Nanopore sequencing, including the barcoding of amplicons by the Native Barcoding Kit (Nanopore, catalog no. SQK-NBD114.24), was performed by the c.ATG/NGS Competence Center Tübingen core facility of the University Hospital Tübingen on a Flongle flow cell. Data processing was performed using the GREPore-seq pipeline (https://github.com/lisiang/GREPore-seq).^47^ SAM files were visualized on Unipro UGENE.

### HTS library preparation for characterization and quantification of editing profiles, off-target sites, and allele-specificity experiments

The peqGOLD Tissue DNA Mini Kit (VWR, catalog no. 13-3096-02) was used to extract genomic DNA after genome editing in accordance with the manufacturer’s instructions. A first PCR amplification was used to amplify the target region by Q5 High-Fidelity DNA Polymerase using 10 ng genomic DNA. To attach Nextera Read adapters to the 5′ end, a second PCR amplification using KAPA HiFi HotStart ReadyMix (Roche, catalog no. KK2601), hybrid primers, and 35 cycles was performed with 1 μL 1:500-diluted template of the first amplification. Subsequently, a third round of PCR amplification of 25 cycles was carried out using KAPA HiFi HotStart ReadyMix to add dual indexes and the Illumina i5 and i7 adapters, with the primers listed in Table S2. The PCR products were then purified with AMPure XP beads following the manufacturer’s protocol, and the purified PCR products were quantified using the Qubit dsDNA Quantification Assay Kits (Thermo Fisher Scientific, catalog no. Q32851). The quantified PCR products were then combined in equal amounts to create a library with a final concentration of 10 μM. The library was sequenced at the c.ATG/NGS Competence Center Tübingen core facility of the University Hospital Tübingen on a MiSeq CRISRPesso2 (https://crispresso.pinellolab.partners.org/submission) was used for alignment, characterization, and quantification of the different editing profiles.^48^ Assessment of the off-target sites was performed by CRISPECTOR (https://github.com/YakhiniGroup/crispector).^49^

### Statistical analysis

Statistical analysis was performed on GraphPad Prism (GraphPad Software) using the two-way t test.

## Data and code availability

The datasets used and/or analyzed during the present study are available from the corresponding author upon reasonable request.

## Acknowledgments

We thank Dr. José Hurst for performing the DLS analysis on the VLPs. We also thank Dr. Andrew Bassett (Wellcome Sanger Institute, UK) for kindly providing the original pKLV2.2-Cas9-TREX2 plasmid. This research was funded by the 10.13039/100017477Usher Syndrome Society translational grant 2022/2023 awarded to S.K. and P.D.A. and the 10.13039/501100001659Deutsche Forschungsgemeinschaft as part of the SPP 2127: Gene and cell-based therapies to counteract neuroretinal degeneration, project no. 498251037, to B.W. We acknowledge support from the Open Access Publication Fund of the 10.13039/501100002345University of Tübingen. The study was conducted in accordance with the ethical principles for research involving human subjects outlined in the Declaration of Helsinki. Fibroblast cell lines were expanded from skin biopsies collected from an adult individual affected by USH2A-related retinal disease upon written informed consent approved by the Ethics Committee of the Medical Faculty of the University of Tübingen. Created in BioRender: Graphical Abstract (https://biorender.com/ewqogmi), Figure 1 (https://biorender.com/95mbv56), and Figure 5C (https://biorender.com/spe41w5).

## Author contributions

Conceptualization, P.D.A.; methodology, P.D.A.; investigation, P.D.A., S. Spaag, S. Shliaga, and A.F.T.; resources, M.R., M.N., K.S., and L.K.; supervision, P.D.A., B.W., and S.K.; funding acquisition, S.K., P.D.A., and B.W.; writing – original draft, P.D.A.; writing – review & editing, S. Spaag, S. Shliaga, A.F.T., M.R., M.N., K.S., L.K., B.W., and S.K.

## Declaration of interests

P.D.A., S.K., and B.W. are inventors of the pending patent WO2023152029, covering parts of the findings described herein.

## Supplemental information

Document S1. Tables S1 and S2 and Figures S1–S9

Table S3. Plasmid sequences

Document S2. Article plus supplemental information
