# Supplementary material for: Single-guide RNA Cas9 and enhanced-deletion Cas9 rescue a recurrent USH2A-related splicing defect
Source: Mol Ther Nucleic Acids. 2025 Mar 21;36(2):102523. doi: 10.1016/j.omtn.2025.102523 (PMC11999688; doi:10.1016/j.omtn.2025.102523)
Supplement: Document S1. Tables S1 and S2 and Figures S1–S9 [file mmc1.pdf]

OMTN, Volume *36*

Supplemental information

Single-guide RNA Cas9 and enhanced-deletion Cas9 rescue a recurrent *USH2A*-related splicing defect

Pietro De Angeli, Salome Spaag, Stefanida Shliaga, Arturo Flores-Tufiño, Malte Ritter, Masoud Nasri, Katarina Stingl, Laura Kühlewein, Bernd Wissinger, and Susanne Kohl

Table S1: Percentage (%) values of correctly spliced *USH2A* transcript achieved in minigene assay and homozygous *USH2A*:c.7595-2144G patient-derived fibroblasts. Results are presented as mean ± SD. The number of biological replicates performed is indicated.

Sample	Percentage of correct *USH2A* transcript (%)	Number of biological replicates
Minigene		
EDCas9_gRNA1_MUTminigene	88.0±1.9%	3
EDCas9_gRNA2_MUTminigene	53.6±11.8%	4
EDCas9_gRNA3_MUTminigene	83.0±12.8%	6
EDCas9_gRNA4_MUTminigene	75.3±9.6%	3
EDCas9_gRNA5_MUTminigene	83.1±8.7%	3
EDCas9_gRNA6_MUTminigene	83.8±15.2%	3
Cas9_gRNA1_MUTminigene	56.6±11.8%	3
Cas9_gRNA2_MUTminigene	34.0±25.3%	3
Cas9_gRNA3_MUTminigene	71.8±30.0%	5
Cas9_gRNA4_MUTminigene	25.1±16.4%	3
Cas9_gRNA5_MUTminigene	56.7±11.2%	3
Cas9_gRNA6_MUTminigene	64.7±10.4%	3
EDCas9_mockgRNA_MUTminigene	7.9±2.2%	2
Cas9_mockgRNA_MUTminigene	5.1±1.0%	3
EDCas9_mockgRNA_WTminigene	100%±0.0%	3
Cas9_mockgRNA_WTminigene	100%±0.0%	3
MUTminigene	3.3±2.0%	2
WTminigene	100%±0.0%	2
Patient-derived fibroblasts		
EDCas9_gRNA1_Fibroblasts	86.6±5.2%	4
EDCas9_gRNA3_Fibroblasts	92.4±4.8%	4
EDCas9_gRNA5_Fibroblasts	85.7±3.7%	3
EDCas9_gRNA6_Fibroblasts	86.3±4.9%	4
Cas9_gRNA1_Fibroblasts	66.8±8.7%	4
Cas9_gRNA3_Fibroblasts	84.6±5.0%	4
Cas9_gRNA5_Fibroblasts	38.2±5.4%	3
Cas9_gRNA6_Fibroblasts	91.8±3.2%	4
Cas9_mockgRNA_Fibroblasts	0.0±.0.%	3
EDCas9_mockgRNA_Fibroblasts	0.0±.0.%	3
Fibroblasts	0.0±.0.%	2

Table S2: List of primers and gRNA oligos used

Name	Sequence (5' – 3')	Use
	**Primers**	
Infusion-minigene-USH2A_F	ATGGGGTACGGGATCACCAGCGCACACACCCTTTCCAATATA	Primers to amplify and clone the *USH2A* fragment into pSPL3
Infusion-minigene-USH2A_R	AGCGGCCGCTCGAGCTCCAGTGAGTGTGTGTATGCGATTCAG	
Infusion-3xFlag-NLS_F	TCACTTTTTTTCAGGTTGGACCGGTGCC	Primers to amplify and clone 3xFLAG-NLS (EDCas9)
Infusion-3xFlag-NLS_R	TCGGCCCGGGGTGCCTCGGAGGCTGCTGGGACTCCGTGGATA	
Infusion-TREX2-Linker_F	TCCGAGGCACCCCGGGCCGAGA	Primers to amplify and clone TREX2 (EDCas9)
Infusion-TREX2-Linker_R	GCTGCCGCCTCCTCCGGCCTCCAGGCTGG	
Infusion-SpCas9FR_F	GGAGGAGGCGGCAGCGACAAGAAGTACAGCATCGGCCTGG	Primers to amplify and clone part of SpCas9 (EDCas9)
Infusion-SpCas9FR_R	AGTGTCAGGGTCAGCACGAT	
USH2A_EX40_F	AATGGATTTGGCAGTGCACATA	Splicing assay in fibroblasts
USH2A_EX41_R	CCTTTTGAAGTGCAGGCTTCTA	
pSPL3seqcDNA_F	TGGACAACCTCAAAGGCACC	Splicing assay by minigene in HEK293T
pSPL3seqcDNA_R	AGTGAATTGGTCGAATGGATC	
pSPL3_SA2_R	ATCTCAGTGGTATTTGTGAGC	pSPL3-specific primer for cDNA synthesis
USH2A-PE40_F	TGCAGTTGCAGGCCAGTTGATT	1st PCR amplification for NGS (gRNA1) and translocation assay
USH2A-PE40_R	TCTGTGATTGGGGGATAAGGCT	
USH2A-PE40-2_F	CCTCTCCAGAATCACACAAG	1st PCR amplification for NGS (gRNA3, gRNA5, and gRNA6)
USH2A-PE40-2_R	CCTCTCTTCCCCAAAGAGAG	

USH2A_PE40_NextAdpt_F	TCGTCGGCAGCGTCAGATGTGTATAAGAGACAGTGCAGTTGCAGGCCAGTTGATT	Primers to add Nextera adapter (gRNA1)
USH2A_PE40_NextAdpt_R	GTCTCGTGGGCTCGGAGATGTGTATAAGAGACAGTCTGTGATTGGGGGATAAGGCT	
USH2A_PE40_2-NextAdpt_F	TCGTCGGCAGCGTCAGATGTGTATAAGAGACAGCCTCTCCAGAATCACACAAG	Primers to add Nextera adapter (gRNA3, gRNA5, and gRNA6)
USH2A_PE40_2-NextAdpt_R	GTCTCGTGGGCTCGGAGATGTGTATAAGAGACAGCCTCTCTTCCCCAAAGAGAG	
USH2A_2178bp_F	AATGCAAACCCCTCAGCCTGCG	Primers to amplify the Nanopore-sequenced fragment
USH2A_2178bp_R	GGCTGGGGCTGAACTTTTGAAGC	
CRISPR-insertcheck-rv2_R	CGCGCTAAAAACGGACTAGC	Primers to check gRNA cloning
pJET1.2_F	CGACTCACTATAGGGAGAGCGGC	Primers to sequence sub-cloned PCR amplicons
pJET1.2_R	AAGAACATCGATTTTCCATGGCAG	
Pklv2.2-PE40_F	AGTTTGGTTAGTACCGGGCCTGCAGTTGCAGGCCAGTTGATT	Primers to clone the lentivirus plasmid containing PE40
Pklv2.2-PE40_R	TCCCCTACCCGGTAGAATTGTCTGTGATTGGGGGATAAGGCT	
VLP-2xNLSP53_F	AGCCAGAACTATCCGATTGTGCAG	Primers to amplify the 2xNLSP53 fragment used for VLP cloning
VLP-2xNLSP53_R	GTCTCGGCCCGGGGTGCCTCGGATCCCCCTGACCCTCCTGGTTTCTT	
VLP-EDCas9_F	TCCGAGGCACCCCGGGCCGAGAC	Primers to amplify the EDCas9 fragment used for VLP cloning
VLP_EDCas9_R	AGTGTCAGGGTCAGCACGAT	
LPA_F	TTCCCTGACTTGGATCAAATGC	Primers to amplify LPA and translocation assay
LPA_R	CATCTGGAGTTGACATGAGCAG	
RHO_F	CTTAGGAGGGGGAGGTCACTTTA	Primers to amplify RHO used to characterized VLPs
RHO_R	GTAGAGCGTGAGGAAGTTGATG	
**gRNA oligos**		
gRNA1_USH2A_F	CACCGTAACTTGTGTGATTCTGGAG	Forward and reverse gRNA oligos used to clone the gRNAs into the backbone editing plasmids. Underlined the sequence of the cloning adapter.
gRNA1_USH2A_R	AAACCTCCAGAATCACACAAGTTAC	
gRNA2_USH2A_F	CACCGAACACCTCTCCTTTCCCA	
gRNA2_USH2A_R	AAACTGGGAAAGGAGAGGTGTTC	
gRNA3_USH2A_F	CACCGTAAAGATGATCTCTTACCTT	
gRNA3_USH2A_R	AAACAAGGTAAGAGATCATCTTTAC	
gRNA4_USH2A_F	CACCGATGATCTCTTACCTTGGGAA	
gRNA4_USH2A_R	AAACTTCCCAAGGTAAGAGATCATC	
gRNA5_USH2A_F	CACCGCTCTTACCTTGGGAAAGGAG	
gRNA5_USH2A_R	AAACCTCCTTTCCCAAGGTAAGAGC	
gRNA6_USH2A_F	CACCGTTAAAGATGATCTCTTACCT	
gRNA6_USH2A_R	AAACAGGTAAGAGATCATCTTTAAC	
gRNA_LPA_F	CACCGTGGACTACATAGTTGTGTGA	
gRNA_LPA_R	AAACTCACACAACTATGTAGTCCAC	

Table S3: Plasmid sequences

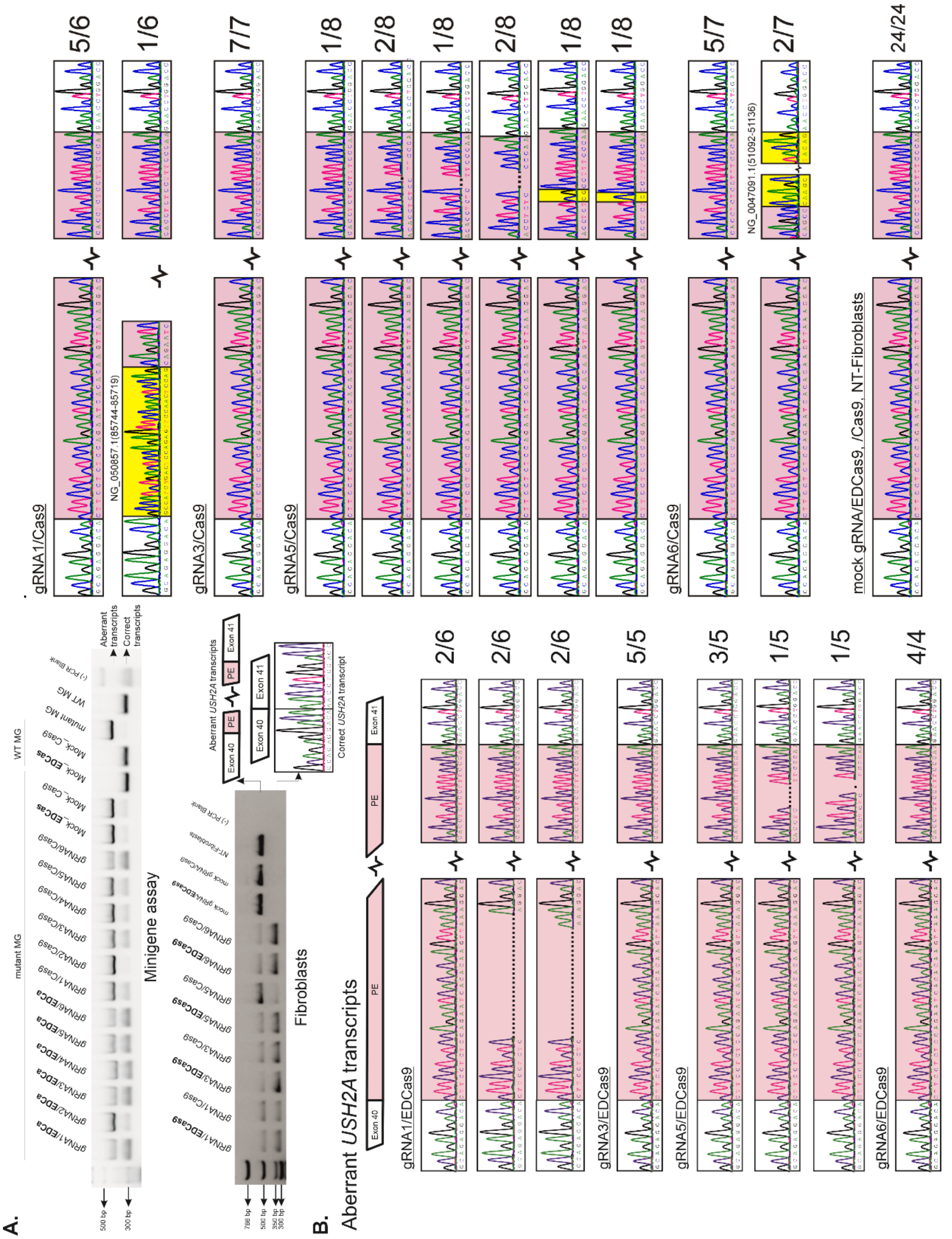

Figure S1: Characterization of the splicing pattern in homozygous *USH2A*:c.7595-2144G patient-derived fibroblasts upon EDCas9 and Cas9-mediated splicing correction. (**A**) Representative agarose gel showing the splicing pattern upon EDCas9 and Cas9 editing in Minigene assay (top) and Fibroblasts (Bottom). The aberrant transcripts are represented by the top band, while the rescued *USH2A* transcript is represented by the bottom band. (**B**) Sequencing characterization of aberrant transcripts upon EDCas9 and Cas9 editing. Individual sub-cloned transcripts were sequenced and aligned to the reference aberrant *USH2A* transcript sequence containing the 152 bp pseudoexon (PE). Some *USH2A* aberrant transcripts show shorter pseudoexon inclusion (dashed lines) upon EDCas9 and Cas9 editing. Single or double nucleotide insertions (in yellow) in the aberrant transcripts are evident for gRNA5/Cas9. Insertion of larger sequence stretches (in yellow) mapped on different genes were detected for gRNA1/Cas9 and gRNA6/Cas9. The ratio of characterized sequences is reported on the right end side of each electropherogram.

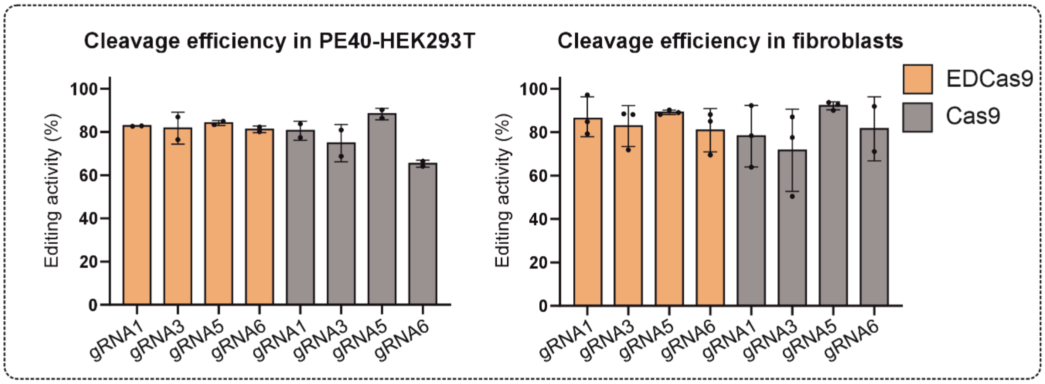

Figure S2: **Gene editing efficiency in PE40-HEK293T (right panel) and homozygous *USH2A*:c.7595-2144G patient-derived fibroblasts (left panel).** Bar graph showing the percentage (%) of gene editing activity. Results are presented as mean (%) ± SD of n=2-3 biological replicates. Difference between EDCas9 and Cas9 is not significant for any gRNA.

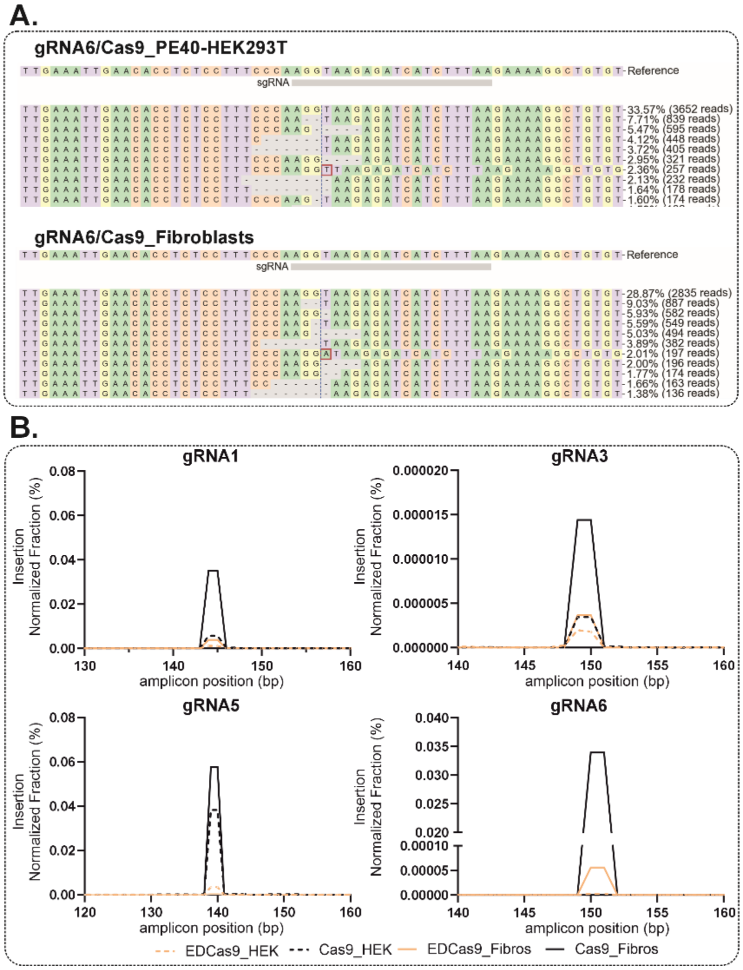

Figure S3: **Mutational profiles generated in PE40-HEK293T and patient-derived *USH2A*:c.7595-2144G fibroblasts. (A)** An exemplary CRISPRESSO allele frequency table for gRNA6 in the two cellular models, illustrating qualitatively similar deletion profiles but with differing relative frequencies. (**B**) Insertion profiling for the lead gRNA1, gRNA3, gRNA5, and gRNA6. The Y axis represents the normalized deletion frequency (%) at each position of the sequenced amplicon (X axis). The data are represented as mean ± SD (n=2-3 independent experiments).

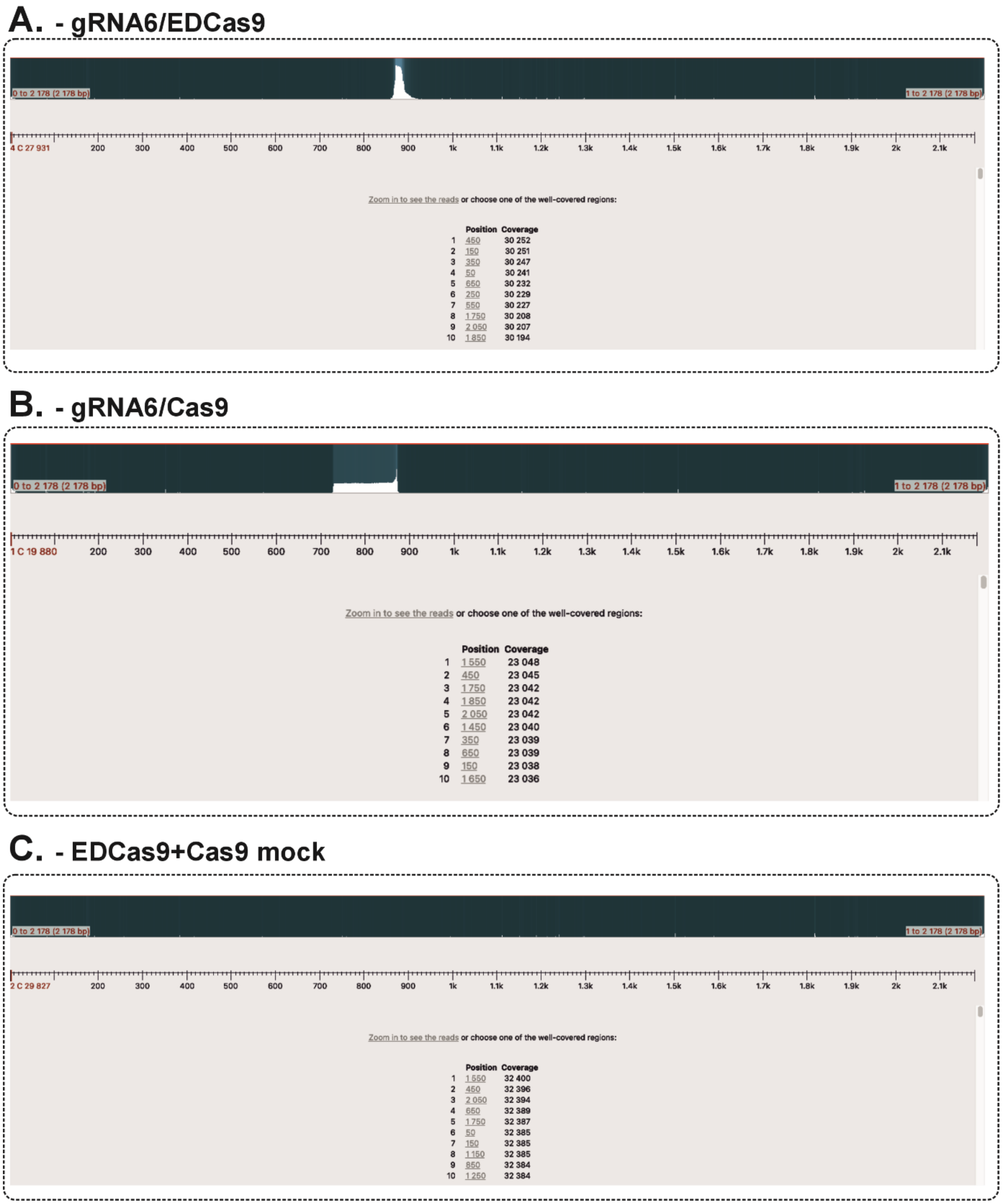

Figure S4: **Visualization of aligned .sam files from long-read Nanopore sequencing of fibroblasts treated with gRNA6/EDCas9 and gRNA6/Cas9.** The position coverage of the top ten nucleotide sites, alongside a graphical representation of aligned amplicons is depicted. The deletion profiles associated with gRNA6/EDCas9 (**A**) and gRNA6/Cas9 (**B**) is highlighted together with the mock-treated control (**C**).

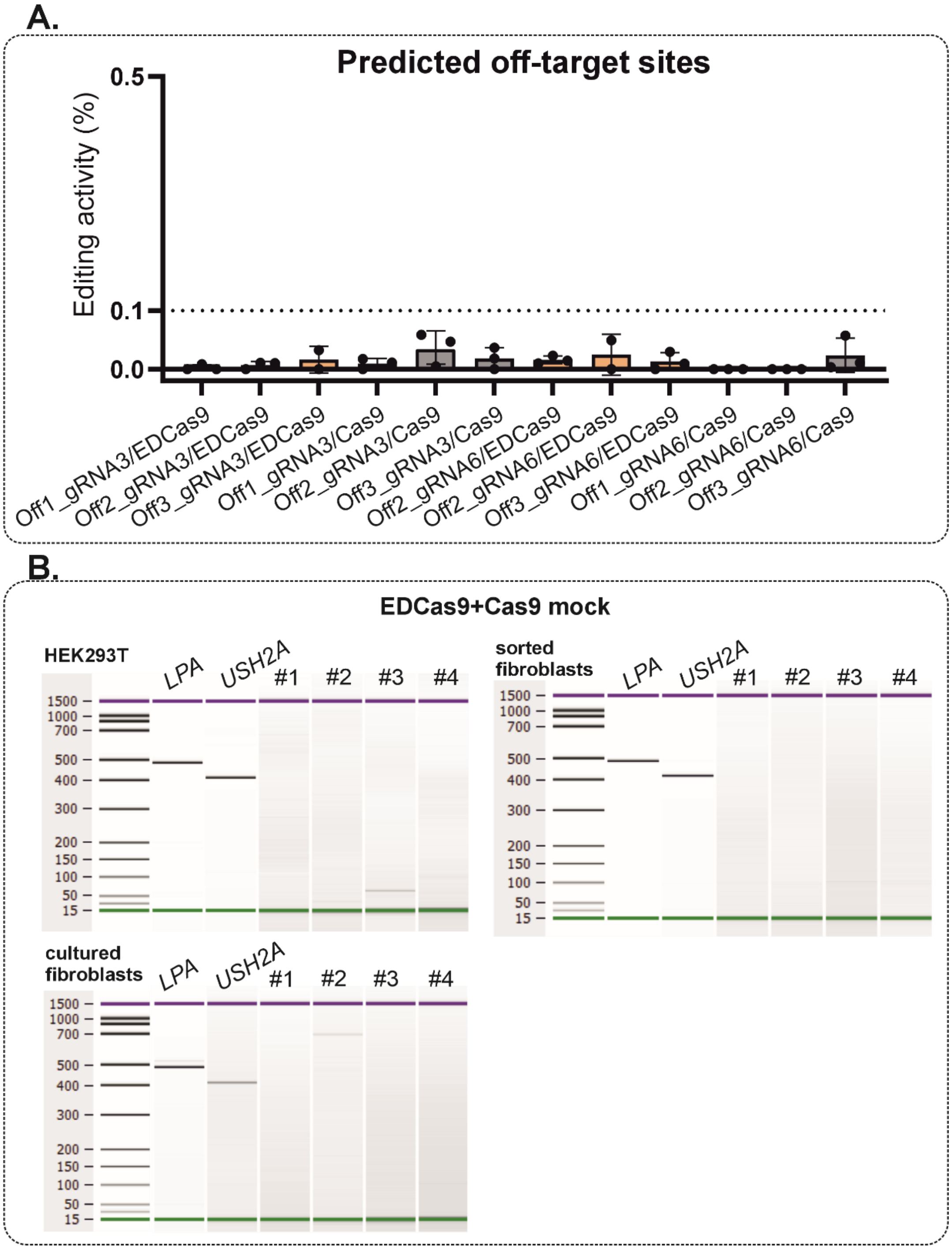

Figure S5: **Off-target assessment in patient-derived fibroblasts (A)** Quantification of the selected off-target sites for gRNA3 and gRNA6 coupled to EDCas9 and Cas9. High-throughput sequencing was used to assess off-target potential. The data was processed on the CRISPRECTOR pipeline. Results are depicted as percentage (%) of editing activity ± standard deviation. Individual data points are shown. The threshold of 0.1% is defined by the pipeline as the threshold for off-target nomination. N=2-3 independent replicates (**B**) Bioanalyzer results of EDCas9+Cas9 mock control samples of the targeted chromosomal translocation assay in HEK293T, sorted fibroblasts (sorted after 72 h post-transfection and processed immediately), and cultured fibroblasts (after sorting, the cells were subcultured for 30 additional days before processing). The lanes indicated with "#" depict the four different chromosomal translocation combinations.

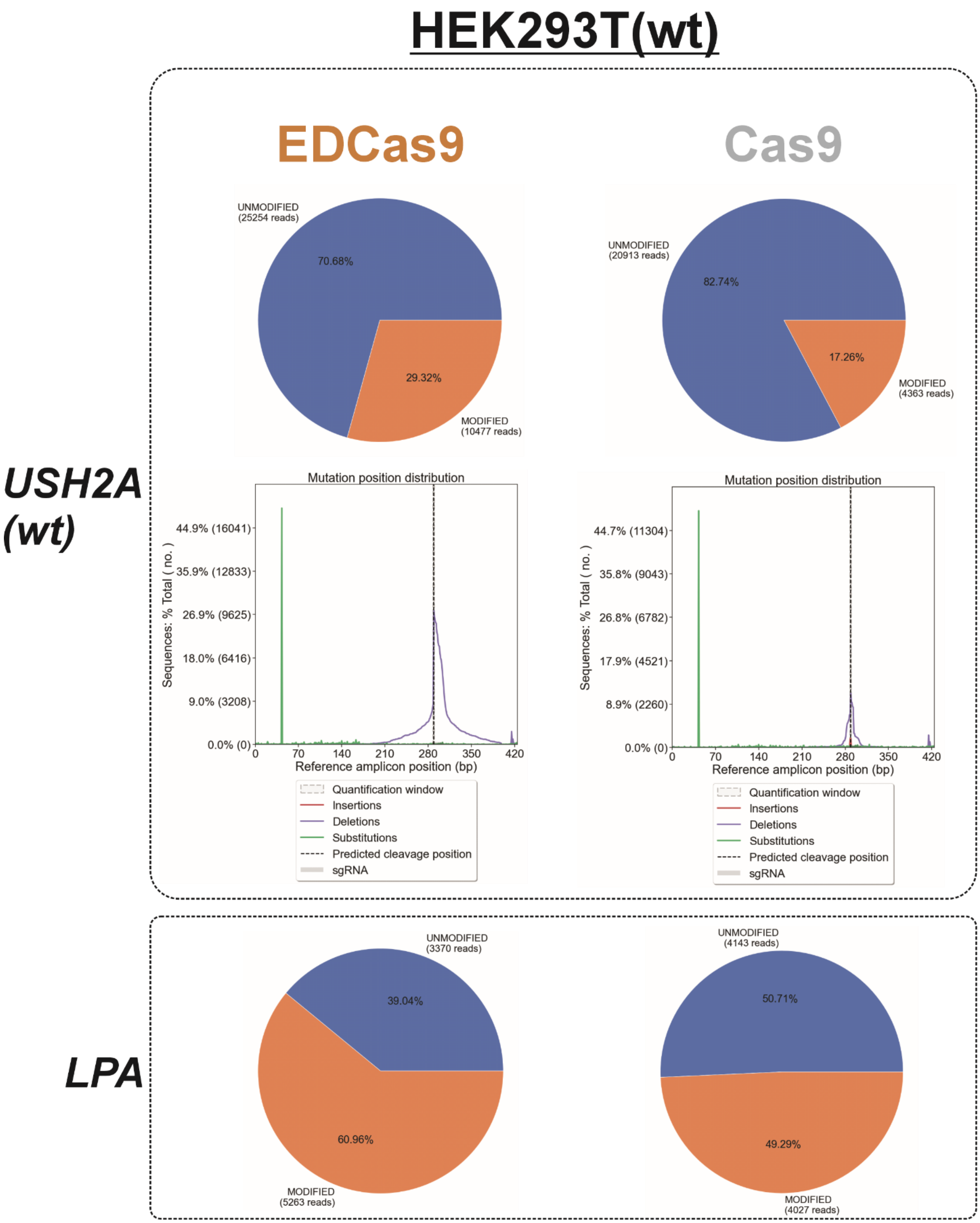

Figure S6: CRISPRESSO analysis of the *USH2A* (wild-type) and *LPA* targeted sequences in HEK293T cells treated with gRNA6(wt) + LPAgRNA/EDCas9 and gRNA6(wt) + LPAgRNA /Cas9. The pie chart represents the fraction of modified (edited) and unmodified (non-edited) reads. Additionally, a mutational profile distribution graph is shown for *USH2A*, highlighting the absence of the ~140 bp deletion for gRNA6/Cas9.

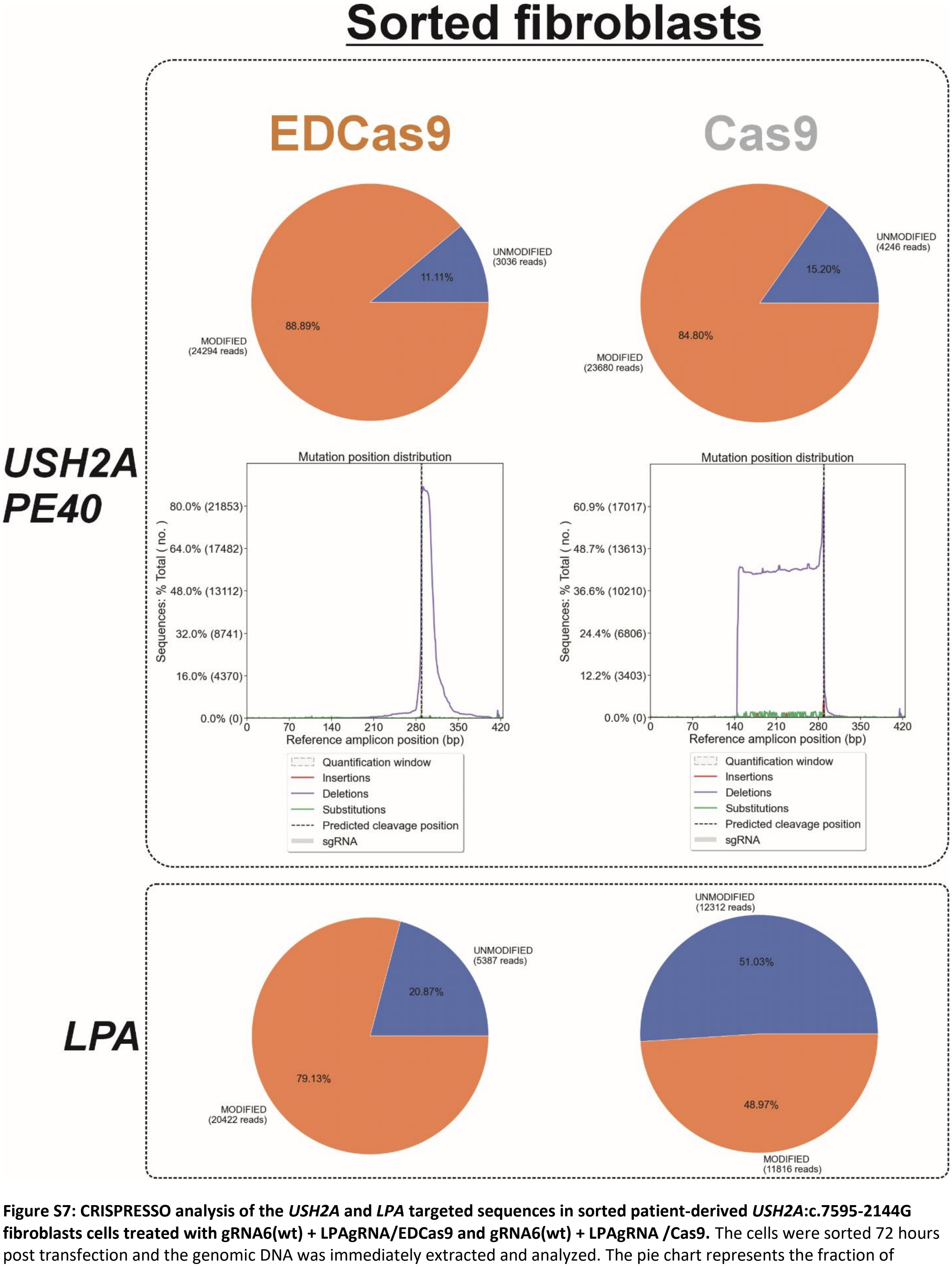

Figure S7: CRISPRESSO analysis of the *USH2A* and *LPA* targeted sequences in sorted patient-derived *USH2A*:c.7595-2144G fibroblasts cells treated with gRNA6(wt) + LPAgRNA/EDCas9 and gRNA6(wt) + LPAgRNA /Cas9. The cells were sorted 72 hours post transfection and the genomic DNA was immediately extracted and analyzed. The pie chart represents the fraction of modified (edited) and unmodified (non-edited) reads. Additionally, a mutational profile distribution graph is shown for *USH2A*, highlighting the presence of the ~140 bp deletion for gRNA6/Cas9.

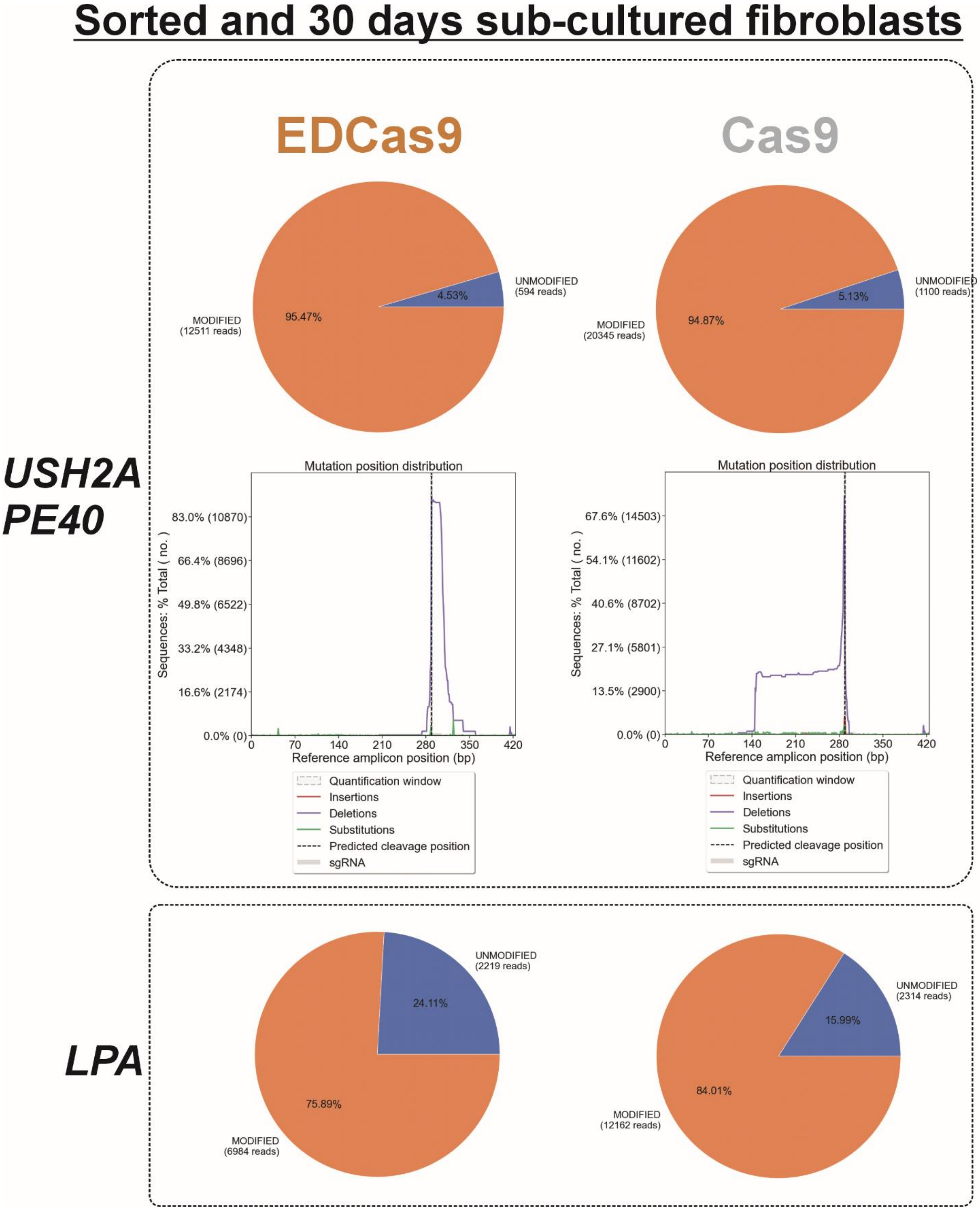

Figure S8: CRISPRESSO analysis of the *USH2A* and *LPA* targeted sequences in sorted and 30 days sub-cultured patient-derived *USH2A*:c.7595-2144G fibroblasts cells treated with gRNA6(wt) + LPAgRNA/EDCas9 and gRNA6(wt) + LPAgRNA /Cas9. The cells were sorted 72 hours post transfection and the genomic DNA was extracted after 30 days of subculturing. The pie chart represents the fraction of modified (edited) and unmodified (non-edited) reads. Additionally, a mutational profile distribution graph is shown for *USH2A*, highlighting the presence of the ~140 bp deletion for gRNA6/Cas9.

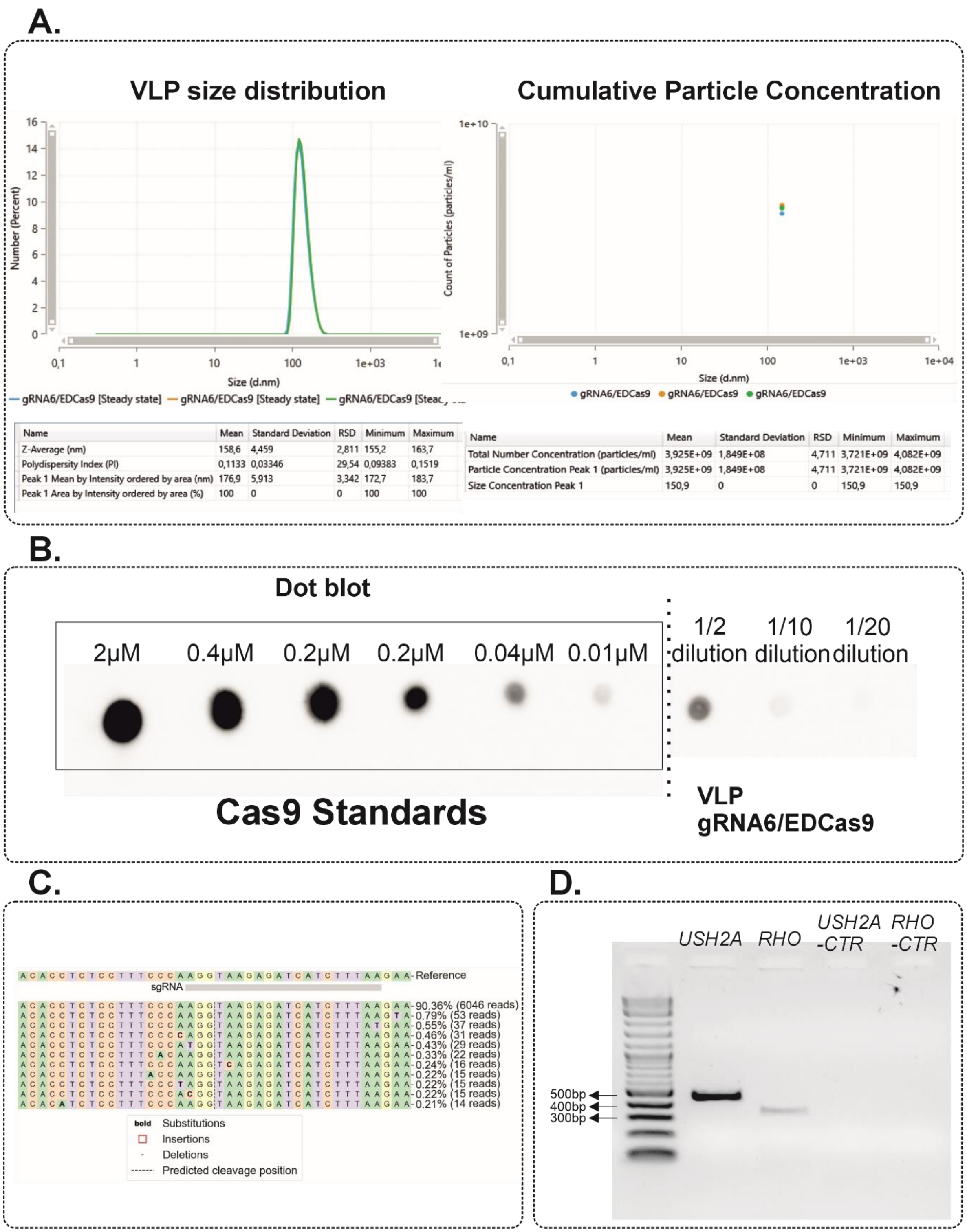

Name	Mean	Standard Deviation	RSD	Minimum	Maximum
Z-Average (nm)	158,6	4,459	2,811	155,2	163,7
Polydispersity Index (PI)	0,1133	0,03346	29,54	0,09383	0,1519
Peak 1 Mean by Intensity ordered by area (nm)	176,9	5,913	3,342	172,7	183,7
Peak 1 Area by Intensity ordered by area (%)	100	0	0	100	100

Name	Mean	Standard Deviation	RSD	Minimum	Maximum
Total Number Concentration (particles/ml)	3,925E+09	1,849E+08	4,711	3,721E+09	4,082E+09
Particle Concentration Peak 1 (particles/ml)	3,925E+09	1,849E+08	4,711	3,721E+09	4,082E+09
Size Concentration Peak 1	150,9	0	0	150,9	150,9

Figure S9: Virus-like particles experiments. (**A**) Characterization of gRNA6/EDCas9 virus-like particles (VLP) by Dynamic Light Scattering (DLS) analysis for assessing particle size distribution and particle concentration. The sample was 1:40 diluted in PBS. (**B**) Dot blot for quantifying encapsulated EDCas9 RNP using an anti-Cas9 antibody. The dashed line indicates where the gel was cropped to arrange the picture (**C**) CRISPRESSO allele frequency table of the *USH2A* amplicon of the corresponding non-treated patient-derived *USH2A*:c.7595-2144G fibroblasts in VLP experiments. (**D**) Agarose gel showing positive amplification of *USH2A* and RHO in the purified VLP preparation, indicating carry over of genomic material from the producer cells. *USH2A* primers: USH2A-PE40-2_F/R – 439 bp fragment. *RHO* primers: RHO_F/R – 323 bp fragment.
